# The lncRNA RP11-142A22.4 promotes adipogenesis by sponging miR-587 to modulate Wnt5β expression

**DOI:** 10.1038/s41419-020-2550-9

**Published:** 2020-06-19

**Authors:** Tongtong Zhang, Hongtao Liu, Rui Mao, Huawu Yang, Yuanchuan Zhang, Yu Zhang, Pengsen Guo, Dafang Zhan, Bin Xiang, Yanjun Liu

**Affiliations:** 1 Medical Research Center, The Third People’s Hospital of Chengdu, The Second Affiliated Hospital of Chengdu, Chongqing Medical University, Chengdu, 610031 Sichuan Province China; 2 Affiliated Hospital of Southwest Jiaotong University, Chengdu, 610036 China; 3 Center for Obesity and Metabolic Diseases, The Third People’s Hospital of Chengdu, The Second Affiliated Hospital of Chengdu, Chongqing Medical University, Chengdu, 610031 Sichuan Province China; 4 Department of Outpatient, The Third People’s Hospital of Chengdu, The Second Affiliated Hospital of Chengdu, Chongqing Medical University, Chengdu, 610031 Sichuan Province China

**Keywords:** Cell biology, Molecular biology

## Abstract

Emerging evidence suggests that long noncoding RNAs (lncRNAs) play essential roles in the regulation of gene expression. However, the functional contributions of lncRNAs to adipogenesis remain largely unexplored. In this study, we investigated global changes in the expression patterns of lncRNAs in visceral adipose tissue and identified RP11-142A22.4 as a significantly upregulated lncRNA. In isolated preadipocytes, knockdown of RP11-142A22.4 inhibited differentiation and reduced C/EBP-α and PPAR-γ expression. Investigations of the underlying mechanisms revealed that RP11-142A22.4 contains a functional miR-587 binding site. Mutation of the binding sites for RP11-142A22.4 in miR-587 abolished the interaction, as indicated by a luciferase reporter assay. Furthermore, RP11-142A22.4 affected the expression of miR-587 and its target gene Wnt5β. Overexpression of miR-587 blocked the inhibitory effect of RP11-142A22.4 on preadipocyte differentiation. Moreover, the downregulation of miR-587 restored preadipocyte differentiation upon inhibition by RP11-142A22.4 silencing. Our results suggest that RP11-142A22.4 can control adipocyte differentiation via the miR-587/Wnt5β signaling pathway and serve as a potential target for obesity treatments.

## Introduction

Obesity has emerged as an epidemic and has become an unprecedented public health challenge. Previous studies have suggested that an increase in adipose tissue is the main cause of obesity and the associated complications^1,2^. Therefore, to understand the functional mechanisms of obesity, it is necessary to study the adipose tissue of obese people.

Over the past years, many crucial obesity-associated genes have been discovered^3^, but their exact functional mechanisms have not yet been fully clarified. Noncoding RNAs (ncRNAs), a group of transcripts showing no protein-coding capacity, have been found to participate in multiple biological and physiological processes^4,5^. Long noncoding RNAs (lncRNAs) are described as highly conserved ncRNAs with a length of more than 200 nucleotides^6^. Several studies have indicated that lncRNAs play a key role in the occurrence and development of obesity^7,8^ and have been considered important regulators of obesity. Despite these findings, the regulatory mechanisms of lncRNAs in adipogenesis are far from understood.

In the present study, we profiled the lncRNA expression patterns in visceral adipose tissue (VAT) of lean people and obese people and found that lncRNA RP11-142A22.4 is the most highly upregulated lncRNA in obese people compared with lean people. Furthermore, we found that RP11-142A22.4 binds to miR-587 to upregulate Wnt5β expression. Together, our results imply that RP11-142A22.4 plays a crucial role in adipogenesis and could serve as a potential therapeutic target for obesity treatment.

## Materials and methods

### Clinical data

VAT samples were collected from 60 patients undergoing laparoscopic hernia repair (in lean [Ln] volunteers) and 40 patients undergoing bariatric surgery (in obese [Ob] subjects) at the Third People’s Hospital of Chengdu, Chengdu, China, between December 2016 and November 2017 (Table S1). This study was approved by the Institutional Ethics Review Board of the Third People’s Hospital of Chengdu (record #: 2018S75; Chengdu, Sichuan, China) and was conducted in accordance with the Chinese ethical guidelines for human genome/gene research.

### LncRNA microarray expression profiling

Total RNA was extracted with TRIzol reagent (Invitrogen, Carlsbad, CA, USA) according to the manufacturer’s instructions. RNA integrity was assessed by an Agilent Bioanalyzer 2100 (Agilent Technologies, Santa Clara, CA, USA). Qualified total RNA was further purified by the RNeasy Mini Kit (QIAGEN, GmBH, Germany) and RNase-Free DNase Set (Qiagen, GmBH, Germany). Probe synthesis and hybridization to Agilent Human 180K lncRNA Microarray v4.0 (Agilent, Santa Clara, CA, USA) were performed by a Gene Expression Hybridization Kit (Agilent, Santa Clara, CA, USA) in a hybridization oven (Agilent, Santa Clara, CA, USA) according to the manufacturer’s instructions. The images were scanned by an Agilent scanner (Agilent, Santa Clara, CA, USA), and the raw data were normalized by a quantile algorithm (Gene Spring Software 11.0, Agilent, Santa Clara, CA, USA). For differentially expressed lncRNAs, we calculated the fold change and the *P*-value (with Student’s *t* test). The threshold for up- and downregulated genes was fold change >2.0 and a *P*-value <0.05. Hierarchical clustering was performed to display the difference in gene expression patterns between the two samples. The obtained lncRNA microarray datasets were deposited into the NCBI Gene Expression Omnibus (GEO) repository under accession number GSE131819.

### Real-time quantitative reverse transcription-polymerase chain reaction

Total RNA was reverse-transcribed into cDNA with random primers using the Transcriptor First Strand cDNA Synthesis Kit (Roche, Penzberg, Germany) following the manufacturer’s instructions. RP11-142A22.4 expression was measured by real-time quantitative reverse transcription-polymerase chain reaction (qRT-PCR) using FastStart Essential DNA Green Master Mix (Roche, Penzberg, Germany) on a Roche LightCycler 480 (Roche, Penzberg, Germany). RNA expression was normalized to GAPDH expression. All quantitative PCRs were conducted in triplicate. Divergent primers, rather than the more commonly used convergent primers, were designed for lncRNAs. We verified the specificity of the PCR primers using BLAST. A single peak in the melt curve indicated that the PCR products were specific.

### Rapid amplification of cDNA ends assay

Total RNA extracted from preadipocytes was subjected to rapid amplification of cDNA ends (RACE) PCR with the SMARTer RACE 5′/3′ Kit (Clontech, Palo Alto, CA, USA) according to the manufacturer’s specifications.

### Prediction of the relationship between lncRNAs and microRNAs

DIANA-LncBase v2 was used to predict microRNA binding sites positioned on lncRNAs^9^. DIANA-LncBase was the first extensive database dedicated to cataloging miRNA–lncRNA interactions and contains the largest collection of experimentally supported and in silico predicted miRNA recognition elements (MREs) on lncRNAs. Each miRNA–lncRNA interacting pair was characterized by a cumulative score, which signifies the interaction strength.

### LncRNA knockdown and microRNA overexpression

Small interfering RNAs (siRNAs) were designed by GenePharma to target lncRNAs (Shanghai, China). Cells were transfected using Lipofectamine 2000 (Invitrogen, CA, USA). After 48 h of siRNA knockdown, lncRNA expression was measured using qRT-PCR. All of the miRNA mimics and inhibitors were synthesized by GeneCopoeia (Rockville, MD, USA).

### LncRNA in vivo precipitation

Biotin-labeled lncRNA probes were synthesized by GenePharma (Shanghai, China) (Table S2). The lncRNA in vivo precipitation (lncRIP) assay was performed as previously described^10^. Preadipocytes were washed with ice-cold phosphate-buffered saline (PBS), fixed using formaldehyde, lysed in co-IP buffer, and sonicated. After centrifugation, the supernatant was added to a probes–M280 Streptavidin Dynabeads (Invitrogen) mixture and further incubated overnight at 30 °C for 12 h. Next, the probes–Dynabeads–lncRNA mixture was washed and incubated with lysis buffer and proteinase K. Finally, TRIzol Reagent (Invitrogen) was added to the mixture for RNA extraction and detection.

### Biotin-coupled miRNA capture

The biotin-coupled miRNA pull-down assay was performed as previously described^11^. Briefly, 3′-end biotinylated miR-587 mimics (Ribio, Guangzhou, China) were transfected into preadipocytes for 48 h before harvest. The cell pellets were incubated with lysis buffer on ice. Then, streptavidin-coated magnetic beads (Life Technologies) were added to the cell lysates to pull down the biotin-coupled RNA complex. The abundance of RP11-142A22.4 in the bound fraction was evaluated by qRT-PCR analysis.

### RNA immunoprecipitation

RNA immunoprecipitation (RIP) experiments were performed using a Magna RIP RNA-Binding Protein Immunoprecipitation Kit (Millipore, Billerica, MA) according to the manufacturer’s instructions. AGO2 antibody (Cell Signaling Technology, Beverly, MA) was used for RIP. Coprecipitated lncRNAs were detected by qRT-PCR.

### Fluorescent in situ hybridization

RNA fluorescence in situ hybridization (RNA-FISH) was performed following the instructions of the probe manufacturer (RiboBio, Guangzhou, China). Preadipocytes were sequentially treated with 70%, 85%, and absolute ethanol and dried at 25 °C. After 0.1% Triton X-100 transmembrane treatment, the cells were incubated with 20 mg/ml RP11-142A22.4 probe overnight at 37 °C. Nuclei were dyed with DAPI. The intracellular localization of RP11-142A22.4 was observed using a TCS SP8 X laser confocal microscope (Leica).

### Plasmid constructs

RP11-142A22.4 was amplified from human genomic DNA and cloned into the pCDNA3.1 vector (Geneseed Biotech Co., Guangzhou, China). Mutations in the miRNA binding sites in the lncRNA sequence were introduced using a Fast Site-Directed Mutagenesis Kit (Takara Bio Inc., Dalian, China). All of the constructs were confirmed by sequencing.

### Dual-luciferase reporter assay

The wild-type RP11-142A22.4 sequence was cloned into the pmiR-RB-Report vector (RiboBio Co., Guangzhou, China), and mutants were generated using site-directed mutagenesis as described above. The mutations were confirmed by sequencing, with vectors containing a mutant sequence used as a control. Preadipocytes were seeded in 96-well plates at a density of 4 × 10^3^ cells per well 24 h before transfection. The cells were then transfected with the wild-type or a mutated reporter vector, and lysates were obtained 24 h post-transfection. The dual-luciferase assay was performed using the Dual-Glo Luciferase Reporter System (Promega, Madison, WI) according to the manufacturer’s protocols.

### Western blot analysis

Proteins were extracted from cultured cells and VAT using RIPA lysis buffer. The protein concentration was determined with a bicinchoninic acid protein assay kit (Sigma). Proteins were separated by 12% SDS-PAGE and transferred to PVDF membranes. After blocking for 1 h, the membranes were incubated with primary antibody at 4 °C overnight. The following antibodies were used: anti-Wnt5β (1:000; Abcam, Cambridge, UK), anti-C/EBP-α and anti-PPAR-γ (1:000; Cell Signaling Technology, Danvers, MA, USA). Membranes were incubated with the appropriate HRP-conjugated secondary antibody at room temperature for 2 h. The immunoreactive bands were visualized using ECL and normalized to GAPDH (the internal control).

### Preadipocyte isolation and differentiation

Preadipocytes from VAT were isolated and cultured following standard protocols^12^. In brief, VAT was digested with collagenase to obtain stromal cells. Stromal cells were separated from mature adipocytes by centrifugation and then incubated in erythrocyte lysis buffer for 10 min at room temperature to eliminate red blood cells. The remaining debris was removed by filtering the cell suspension through a 70-μm nylon filter and centrifuging the filtrate. Pelleted preadipocytes were plated in basal medium consisting of DMEM/F-12 (Gibco, Carlsbad, CA) supplemented with 10% fetal calf serum (FCS) and incubated for 16–18 h. After incubation, attached cells were washed thoroughly with warm PBS, removed from plates with trypsin, resuspended, and counted.

We then induced preadipocyte differentiation by incubating cells (at 2 days post-confluence; defined as day 0) in induction medium supplemented with 5 μg/ml insulin (MedChem Express), 1 μM dexamethasone, and 0.5 mM methylisobutylxanthine for 48 h. The induction medium was replaced with DMEM containing 5 μg/ml insulin, and cells were incubated for an additional 48 h. Then, cells were incubated in DMEM containing 10% FBS until most of the cells had differentiated into mature adipocytes with abundant lipid droplets (day 6).

### Oil Red O staining

Intracellular lipid deposits were visualized by staining with Oil Red O. At different time points after the medium was changed, wild-type preadipocytes were stained with 30% Oil Red O in isopropanol for 60 min. The lipid deposits in differentiated cells were identified using light microscopy. To quantify the degree of preadipocyte differentiation, spectrophotometric quantification of the stain was performed by dissolving the stained Oil Red O with isopropanol for 5 min. The absorbance of the extract was measured at 540 nm.

### Statistical analyses

All statistical analyses were performed with SPSS v20.0 (SPSS Inc., Chicago, IL). Student’s *t* test was used to compare the two groups. The chi-square test was used to identify significant correlations between RP11-142A22.4 expression and clinical-pathological features in obese patients. Multivariate logistic regression was performed to estimate odds ratios for the potential predictors of 1-year nonremission. *P* < 0.05 was considered to indicate statistical significance.

## Results

### Expression profile of lncRNA in VAT

**Fig. 1:**
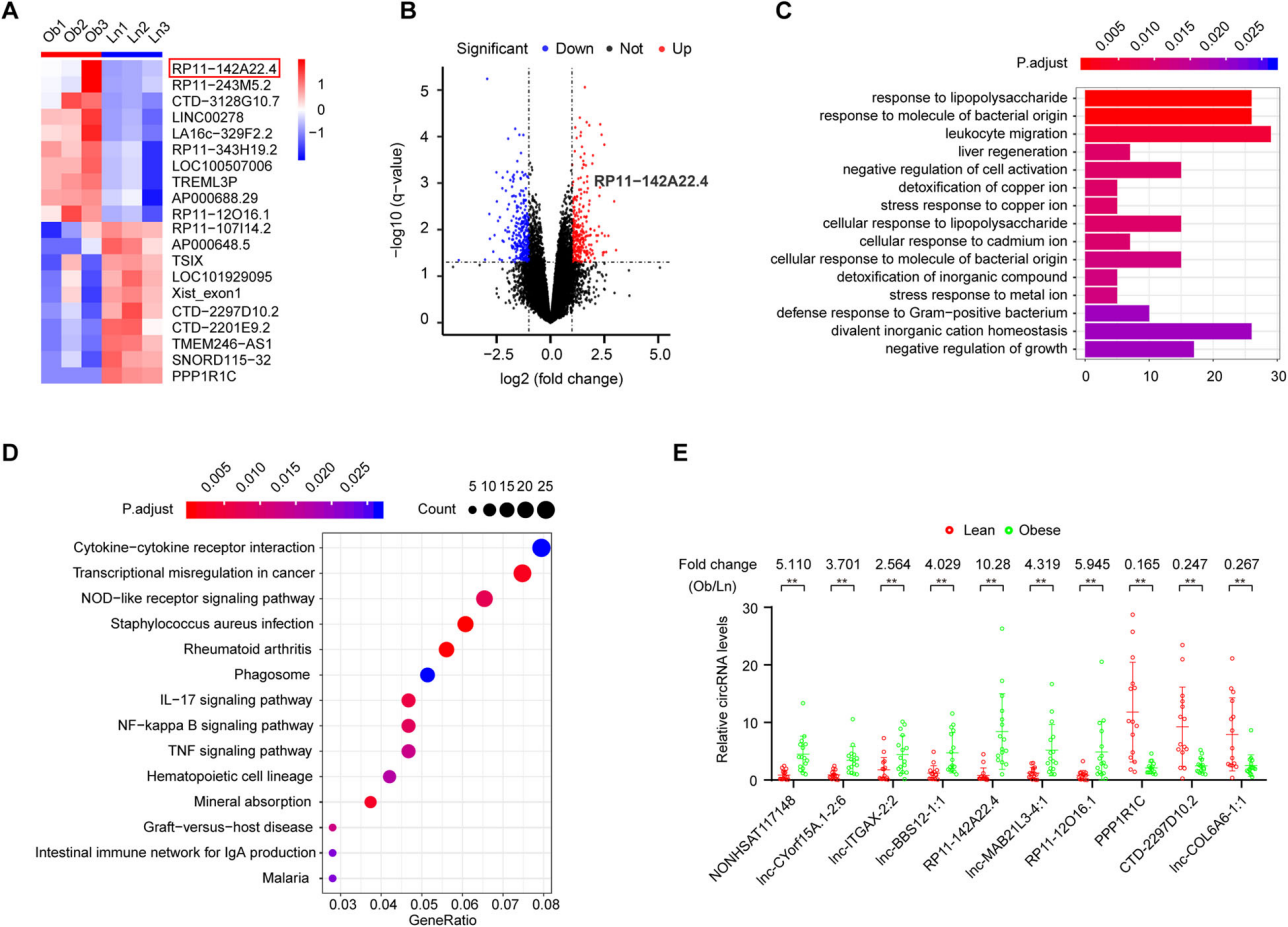
Identification of differentially expressed lncRNAs in obese patients. **a** Clustered heatmap of the differentially expressed lncRNAs in VAT samples. Upregulated lncRNAs are shown in red and downregulated lncRNAs are shown in green. **b** Volcano plots comparing lncRNA expression between obese and lean patients. The red dots represent the significantly differentially expressed lncRNAs (fold-change ≥1.5 and *P* < 0.05). **c** Genomic origins of the differentially expressed lncRNAs in obese patients. **d** Top 15 classes of KEGG pathway enrichment terms. **e** Top 15 classes of disease enrichment terms. **f** The differential expression of 10 lncRNAs was validated in 10 adipose from obese and 10 adipose from lean using RT-qPCR. Data are presented as means ± SD; significant difference was identified with Student’s *t* test. *P < 0.05; **P < 0.01; ns, not significant.

We identified 331 lncRNAs with significantly differential expression in VAT between lean and obese people (*P* < 0.05). Of these lncRNAs, 153 were upregulated and 178 were downregulated. Unsupervised hierarchical clustering analysis results of the expression of the top 20 differentially expressed lncRNAs are shown in a heat map (Fig. 1a). Significantly differentially expressed lncRNAs were identified using volcano plot filtering (Fig. 1b). Both functional enrichment analysis and KEGG signaling pathway analysis also suggested that the abnormal expression of lncRNAs was associated with lipopolysaccharide syndrome and its related pathway (Fig. 1c, d). We also constructed a network containing lncRNA-miRNA-mRNA pathways, which might be responsible for the regulation of the biological mechanisms underlying obesity (Supplementary Fig. 1).

### Identification and characterization of RP11-142A22.4

**Fig. 2:**
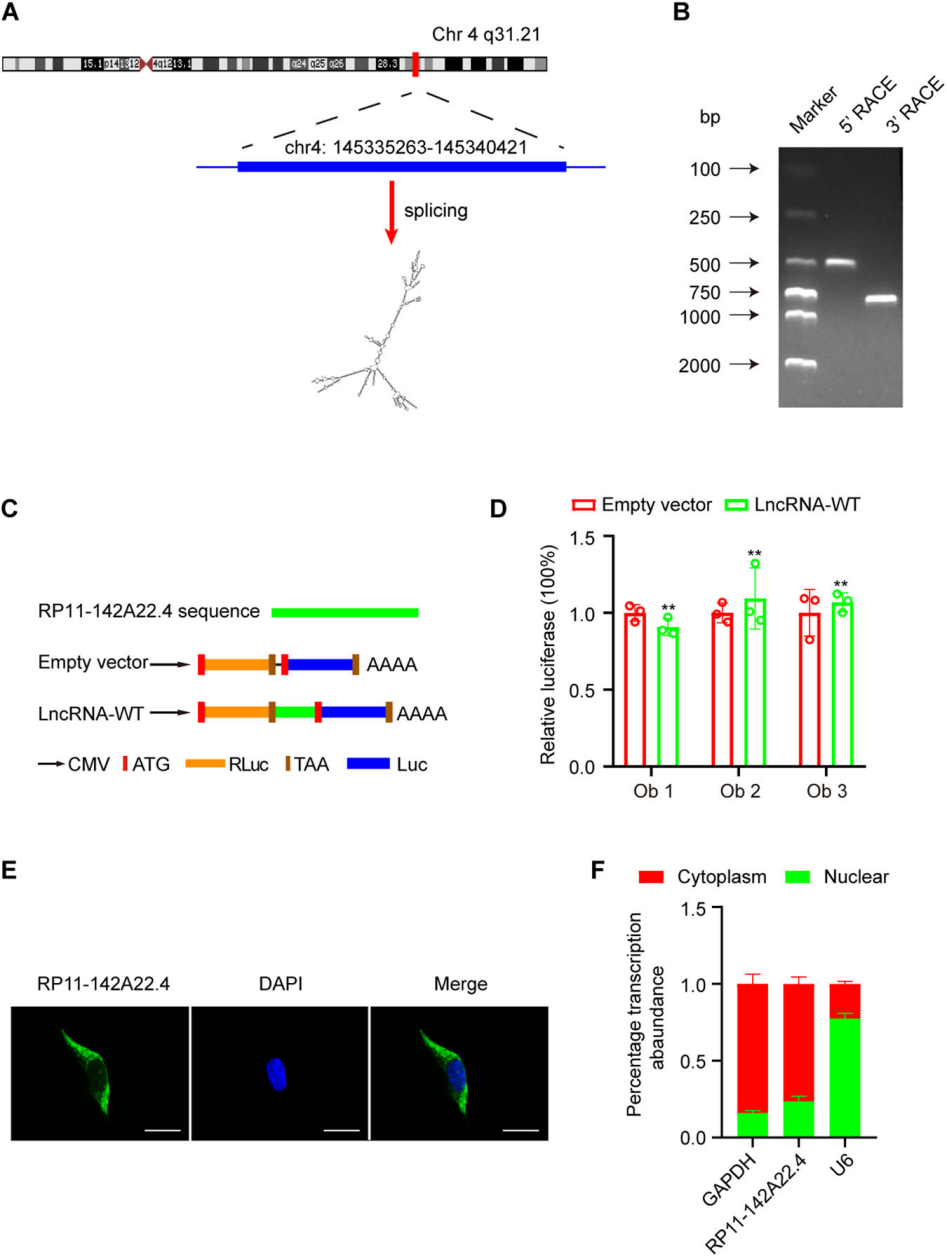
Characterization of RP11-142A22.4 in preadipocytes. **a** The genomic location of the RP11-142A22.4. **b** The full-length RP11-142A22.4 was achieved using 5’ and 3’ rapid amplification of cDNA end reactions. The bands (arrows) for RP11-142A22.4 are indicated. **c** RP11-142A22.4 sequences were cloned between Rluc and Luc reporter genes with independent start and stop codons. **d** The relative luciferase activity of Luc/Rluc in the above vectors was tested. **e** RNA FISH for RP11-142A22.4. Nuclei were stained with DAPI. Scale bar = 20 µm. **f** Relative RP11-142A22.4 expression levels in nuclear and cytosolic fractions of preadipocytes. Scale bar = 20 µm. Data are presented as means ± SD; significant difference was identified with Student’s *t* test. *P < 0.05; **P < 0.01; ns, not significant.

After examining the obtained microarray data, we focused on the top 10 differentially expressed lncRNAs (i.e., with the largest fold changes) for further examination (Table S3). These lncRNAs were further examined using qRT-PCR; 10 obese and 10 lean VAT samples were examined (Fig. 1e). The most significantly differentially expressed lncRNA was RP11-142A22.4, and based on the human reference genome GRCh37/hg19, we found that RP11-142A22.4 (chr4: 145335263-145340421) is located on chromosome 4q31 (Fig. 2a). The sequence of RP11-142A22.4 was confirmed by Sanger sequencing (Supplementary Fig. 2).

To determine both ends of this transcript, we performed 5′ and 3′ RACE assays in preadipocytes (Fig. 2b). We then examined the coding potential of RP11-142A22.4 using NONCODE databases and LncRBase. These data consistently indicated that RP11-142A22.4 had no ability to encode a protein. Moreover, we used a dual-luciferase vector system to test the putative translation ability of RP11-142A22.4. Full-length RP11-142A22.4 sequences were cloned between the Rluc and Luc reporter genes. Rluc and Luc reporters with independent start and stop codons were directly connected in the empty vector (Fig. 2c). As empty vectors, full-length RP11-142A22.4 could not induce Luc activity (Fig. 2d). To assay the subcellular distribution of RP11-142A22.4, we performed RNA-FISH and qRT-PCR. Our results show that most of the RP11-142A22.4 transcripts were located in the cytoplasm (Fig. 2e, f and Supplementary Fig. 3).

### RP11-142A22.4 upregulation predicts aggressive clinical-pathological characteristics

**Fig. 3:**
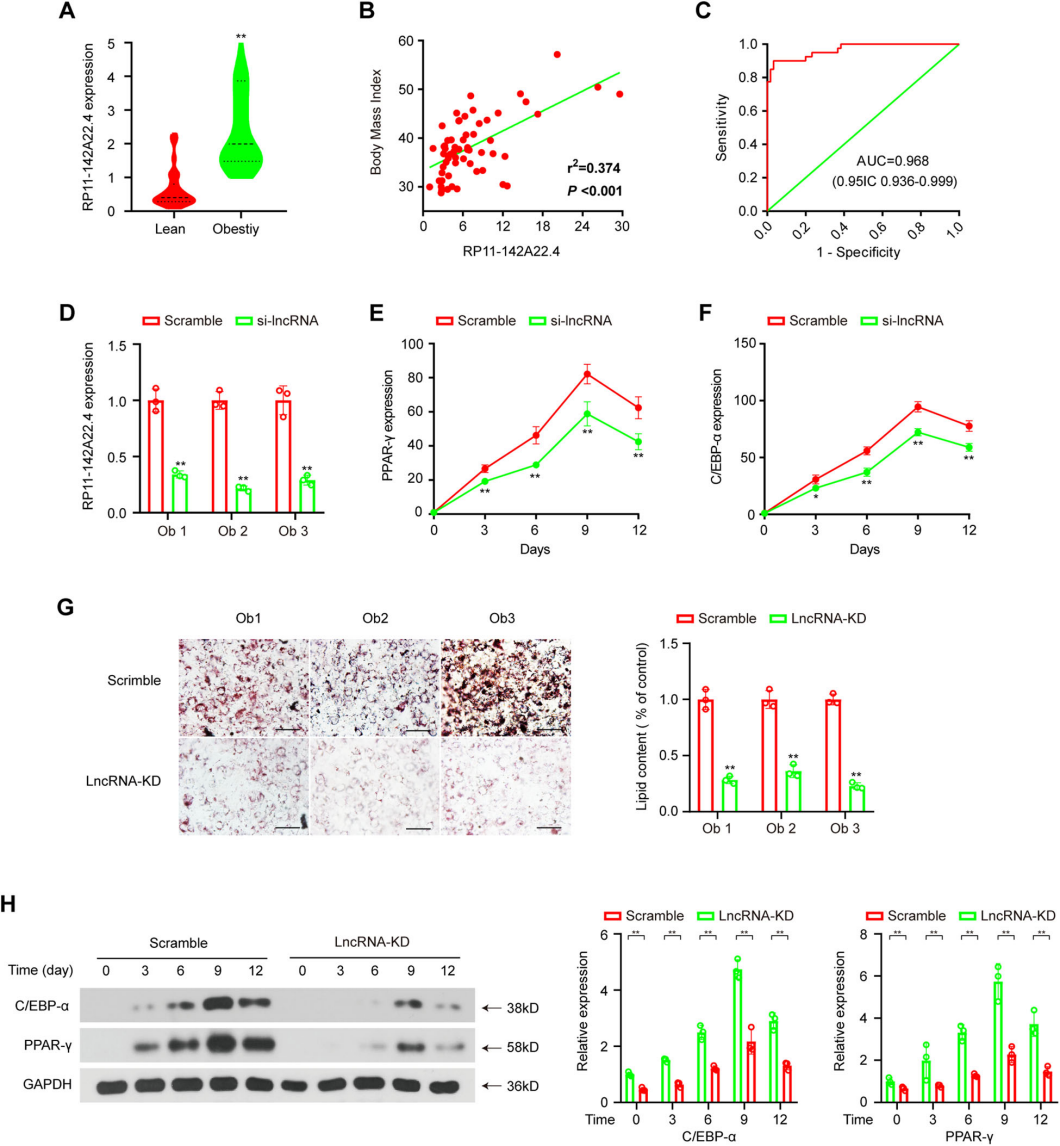
RP11-142A22.4 upregulation predicts aggressive clinical-pathological characteristics and regulates preadipocyte differentiation in vitro. **a** RP11-142A22.4 expression in adipose tissues from 60 obese patients and 40 lean patients. **b** Pearson correlation between RP11-142A22.4 expression and BMI in the adipose tissues of 60 obese patients. **c** ROC curve for RP11-142A22.4 that indicates a diagnostic value for this lncRNA in obese patients. **d** Valuation of RP11-142A22.4 expression after siRNA treatment using qRT-PCR. **e**, **f** C/EBP-α and PPAR-γ expression was quantified using qRT-PCR after RP11-142A22.4 knockdown during preadipocytes differentiation. **g** Oil red O staining of differentiated preadipocytes after transfection with RP11-142A22.4 specific siRNA versus scramble controls. Scale bar = 100 µm. **h** C/EBP-α and PPAR-γ expression was quantified using western blot assay after RP11-142A22.4 knockdown during preadipocytes differentiation. Data are presented as means ± SD; significant difference was identified with Student’s *t* test. *P < 0.05; **P < 0.01; ns, not significant.

As RP11-142A22.4 expression was significantly increased in VATs from obese patients, we next analyzed whether RP11-142A22.4 upregulation was correlated with the prognosis of these patients. We used qRT-PCR to quantify RP11-142A22.4 expression in a cohort of 60 obese patients for whom prognostic data were available. RP11-142A22.4 expression was significantly upregulated in the VATs from obese patients compared to the VATs from lean patients (Fig. 3a). We also analyzed the association between RP11-142A22.4 expression and the clinical-pathological status of obese patients. RP11-142A22.4 expression was positively correlated with body mass index (BMI) (*P* < 0.01) in obese patients (Fig. 3b and Table 1). Furthermore, ROC analysis was performed with an RP11-142A22.4 cut-off ≥2.298 and showed a high diagnostic performance, as reflected by the Youden index (sensitivity, 90.0%; specificity, 96.7%; Fig. 3c).

**Table 1:** RP11-142A22.4 expression in obese sample.

Clinical or molecular feature	RP11-142A22.4 (30) over-expression	RP11-142A22.4 (30) down-expression	*P*
Age (y)	25 ± 3.74	30.9 ± 5.6	*1*
Gender (M/F)	7/23	7/23	*—*
BMI (kg/m^2^)	41.1 ± 6.3	35.3 ± 4.4	*0.001*
Systolic BP (mm Hg)	127.6 ± 10.9	132.0 ± 17.5	*0.256*
Diastolic BP (mm Hg)	78.1 ± 7.4	84.5 ± 12.7	*0.022*
Diabetes	13.3% (4/30)	16.6% (5/30)	*0.723*
Hyperuricemia (HUA)	26.7% (8/30)	13.3% (4/30)	*0.203*
Hyperlipemia (HLP)	53.3% (16/30)	63.3% (19/30)	*0.441*
Obstructive sleep apnea syndrome (OSAS)	76.6% (23/30)	53.3% (16/30)	*0.060*
Hyperinsulinemia (HINS)	6.6% (2/30)	16.6% (5/30)	*0.235*

Italic value stands for specific *p* values.

### **RP11-142A22.4 regulates preadipocyte differentiationin vitro**

To explore the role of RP11-142A22.4 in preadipocytes, we successfully knocked down RP11-142A22.4 in preadipocytes from obese patients (Fig. 3d). Oil Red O staining indicated that RP11-142A22.4 knockdown downregulated preadipocyte differentiation (Fig. 3g). The expression levels of C/EBP-α and PPAR-γ, which are biomarkers of preadipocyte differentiation^13,14^, were decreased in RP11-142A22.4 knockdown cells, as quantified by qRT-PCR and western blot analysis (Fig. 3e, f, h and Supplementary Fig. 4). Similar results were obtained using preadipocyte from the subcutaneous adipose tissue (SAT) samples of obese patients (Supplementary Fig. 5).

### RP11-142A22.4 might function as a sponge for miR-587

In addition to epigenetic regulation in the nucleus, lncRNAs can regulate target gene expression by functioning as competing endogenous RNAs (ceRNAs) for specific miRNAs in the cytoplasm^15^. RP11-142A22.4 was identified as a cytoplasm-enriched abundant lncRNA in preadipocytes. The potential targets of RP11-142A22.4 were predicted using a bioinformatics database (DIANA-LncBase v2). Using this approach, we identified 10 potential interacting miRNAs and constructed an RP11-142A22.4–miRNA–mRNA network (Supplementary Fig. 1 and Table S4).

**Fig. 4:**
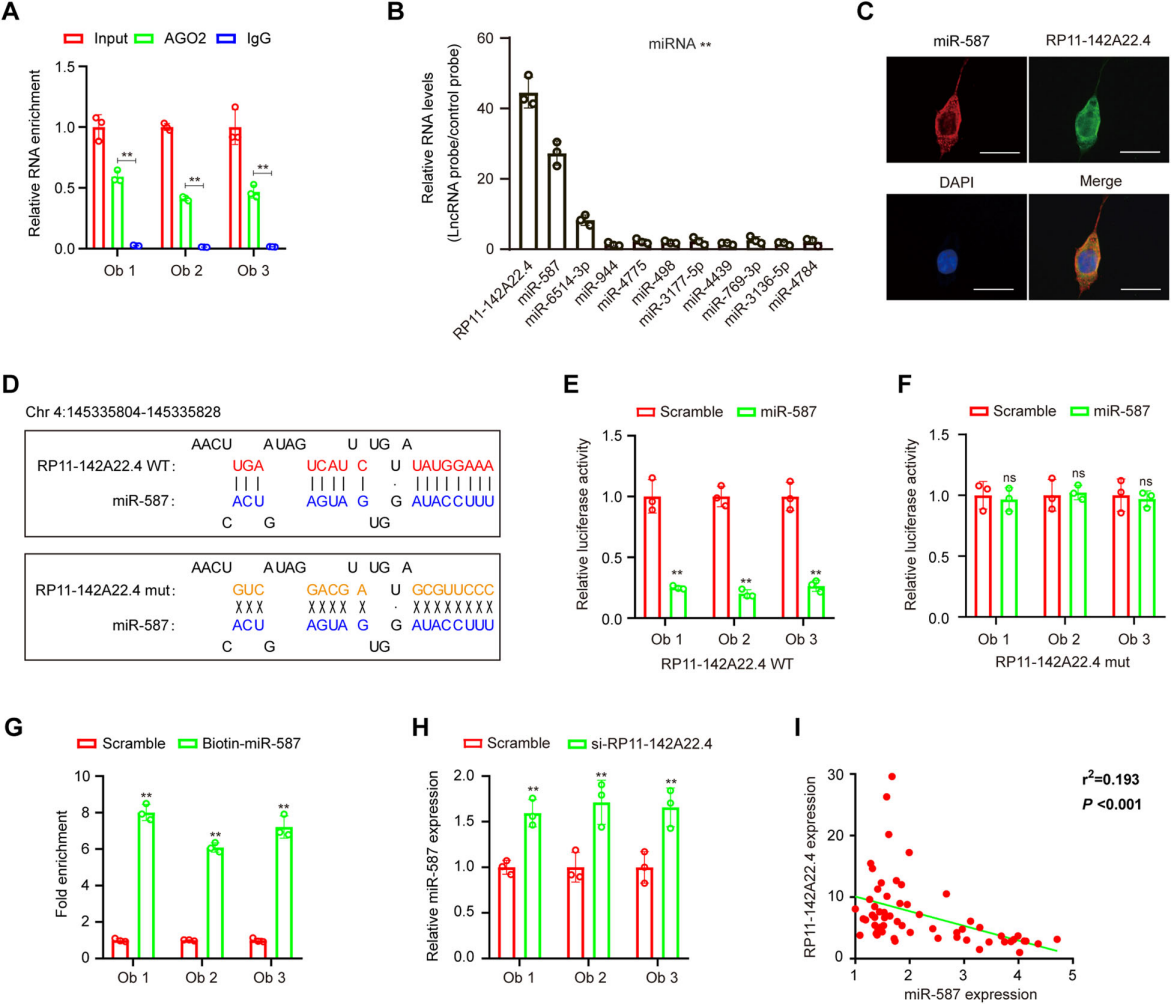
RP11-142A22.4 acts as a miRNAs sponge for miR-587. **a** RIP experiments were performed using an antibody against AGO2 on extracts from preadipocytes. **b** LncRIP was performed using a RP11-142A22.4-specific probe and control probe in preadipocytes from obesity patients. The enrichment of RP11-142A22.4 and microRNAs was detected by RT-qPCR and normalized to the control probe. **c** Co-localization between RP11-142A22.4 and miR-587 was observed by RNA in situ hybridization in preadipocytes. Nuclei were stained with DAPI. Scale bar = 20 µm. **d** Schematic showing the predicted miR-587 sites in RP11-142A22.4. A Luciferase assay where preadipocytes were co-transfected with a scrambled control, miR-587 mimic, and a luciferase reporter plasmid containing wild-type RP11-142A22.4 (RP11-142A22.4-WT) (**e**) or mutant RP11-142A22.4 (RP11-142A22.4-mut) (**f**). **g** Expression of miR-587 was analyzed using RT-qPCR following RP11-142A22.4 knockdown. **h** RT-qPCR showed the level of RP11-142A22.4 in the streptavidin-captured fractions from the preadipocytes lysates after transfection with biotinylated miR-587 or control RNA. **i** Pearson correlation between RP11-142A22.4 expression and miR-587 expression in the adipose tissues of 60 obese patients using qRT-PCR. Data are presented as means ± SD; significant difference was identified with Student’s *t* test. *P < 0.05; **P < 0.01; ns, not significant.

To further explore RP11-142A22.4 associations, a lncRIP assay with antibodies against Argonaute 2 (AGO2) was chosen because AGO2 plays an important role in miRNA-induced RNA silencing^16^. The results show that the anti-AGO2 antibody significantly enriched RP11-142A22.4 (Fig. 4a), suggesting that RP11-142A22.4 acts as a binding platform for AGO2 and miRNAs. Additionally, RP11-142A22.4-associated miRNAs were purified by lncRIP with specific probes targeting RP11-142A22.4. The results show that RP11-142A22.4 and miR-587 were both enriched in the examined preadipocytes (Fig. 4b and Supplementary Fig. 6). This association was further confirmed using RNA-FISH, which showed that RP11-142A22.4 and miR-587 are colocalized in preadipocytes (Fig. 4c).

To examine the predicted RP11-142A22.4 binding sites (Fig. 4d), a dual-luciferase assay was performed. The results show that high binding affinity exists between RP11-142A22.4 and miR-587 (Fig. 4e). Furthermore, miR-587 reduced luciferase reporter activity by >50% compared to the control (Fig. 4e). After mutating the miR-587 target sites in the luciferase reporter, no significant change in luciferase activity was observed following transfection with miR-587 and the luciferase reporter (Fig. 4f). Furthermore, a pull-down assay using biotin-coupled miR-587 showed obvious enrichment of RP11-142A22.4 compared with the controls (Fig. 4g).

To verify whether RP11-142A22.4 regulates the expression of miR-587, miR-587 expression was examined following RP11-142A22.4 overexpression or knockdown in preadipocytes. After knockdown of RP11-142A22.4, the expression of miR-587 was significantly upregulated (Fig. 4h). We also found a significant negative correlation between RP11-142A22.4 and miR-587 expression in VAT (Fig. 4i). Thus, our results suggest that RP11-142A22.4 knockdown suppresses preadipocyte differentiation by reducing the functionality of miR-587.

### Knockdown of RP11-142A22.4 inhibits preadipocyte differentiation via the miR-587/Wnt5β pathway

**Fig. 5:**
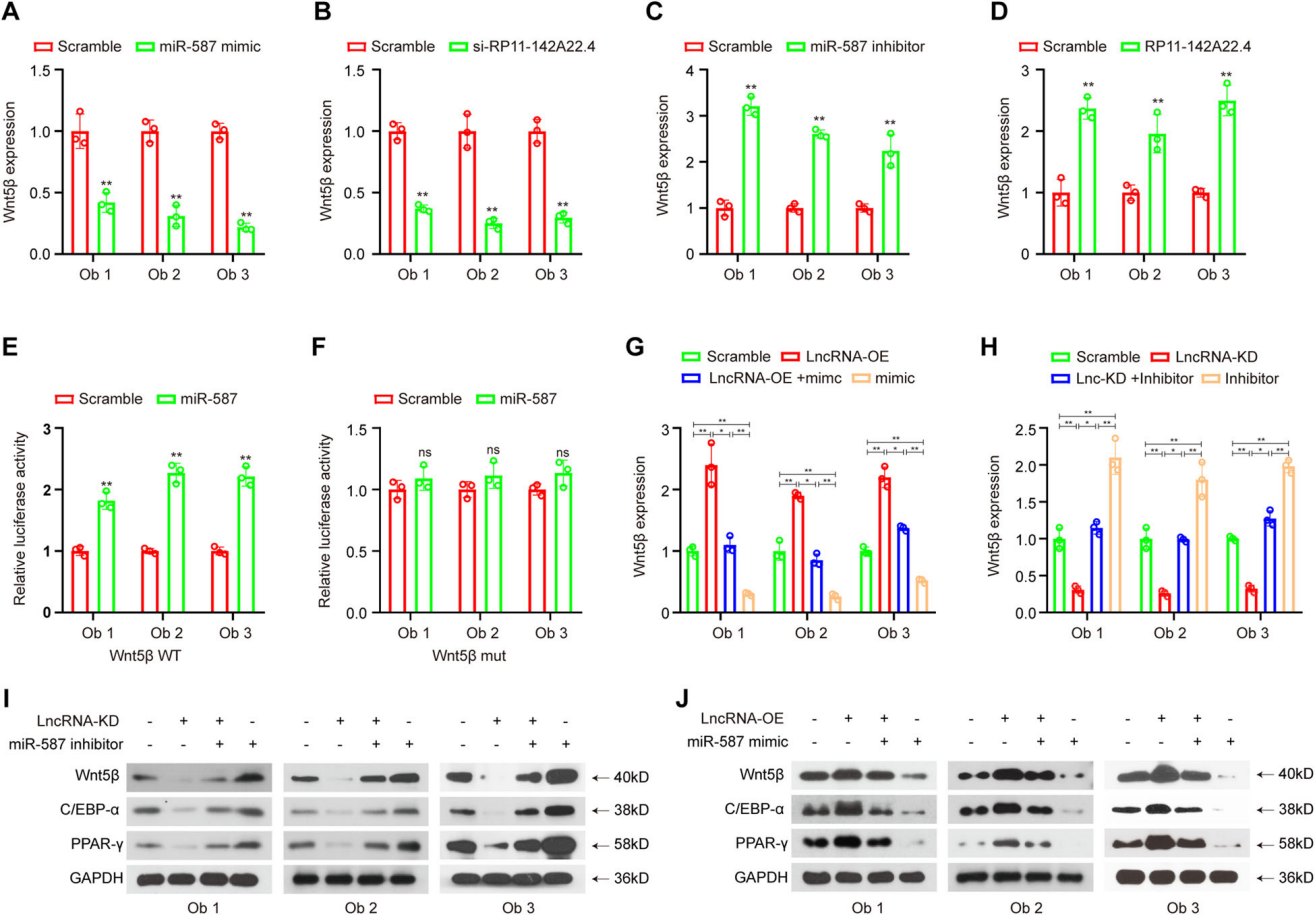
RP11-142A22.4 promotes adipogenesis through the miR-587/Wnt5β pathway. qRT-PCR quantification of Wnt5β expression after miR-587 knockdown (**a**) or RP11-142A22.4 overexpression (**b**). Wnt5β expression after transfection with RP11-142A22.4-specific siRNAs (**c**) or with miR-587 mimic was quantified with qRT-PCR (**d**). Luciferase assay where preadipocytes were co-transfected with a scrambled control, miR-587 mimic, and a luciferase reporter plasmid containing either wild-type Wnt5β (Wnt5β-wt) (**e**) or a Wnt5β construct with mutated miR-587 binding sites (Wnt5β-mut) (**f**). **g**, **h** Reversion assays using vectors overexpressing or knocking down RP11-142A22.4, as well as miR-587 mimics or inhibitors. Data are presented as means ± SD; significant difference was identified with Student’s *t* test. *P < 0.05; **P < 0.01; ns, not significant.

The bioinformatics method TargetScan predicted that Wnt5β is a possible downstream gene of miR-587 (Supplementary Fig. 7). Thus, we hypothesized that RP11-142A22.4 induces preadipocyte differentiation by protecting the differentiation promoting factor Wnt5β from downregulation by miR-587. To test this hypothesis, we overexpressed miR-587 mimics and measured the expression of their respective targets via qRT-PCR (Table S2). Following mimic transfection into preadipocytes, Wnt5β was found to exhibit a significant decrease in gene expression (Fig. 5a). Furthermore, RP11-142A22.4 knockdown significantly reduced Wnt5β expression (Fig. 5b), while RP11-142A22.4 overexpression or miR-587 inhibition increased Wnt5β expression (Fig. 5c, d).

To further examine Wnt5β, the 3′ untranslated region (UTR) of Wnt5β was cloned into a luciferase vector, and the effect of miR-587 in the presence or absence of RP11-142A22.4 was examined. Our data showed that no significant change in luciferase activity was observed following transfection with RP11-142A22.4 compared with the control (Supplementary Fig. 8). In preadipocytes overexpressing miR-587, the luciferase reporter activity was enhanced in those containing the wild-type Wnt5β 3′UTR compared to the control but not in those containing a mutated Wnt5β 3′UTR (Fig. 5e, f). However, in cells with RP11-142A22.4 knockdown and miR-587 inhibition, the expression levels of Wnt5β and downstream C/EBP-α and PPAR-γ expression^17,18^ were significantly rescued (Fig. 5g, i and Supplementary Fig. 9). Furthermore, miR-587 upregulation significantly reduced Wnt5β levels further upon RP11-142A22.4 knockdown (Fig. 5h, j and Supplementary Fig. 10). These data suggest that RP11-142A22.4 induces preadipocyte differentiation by interacting with miR-587 within the RP11-142A22.4/miR-587/Wnt5β axis.

**Fig. 6:**
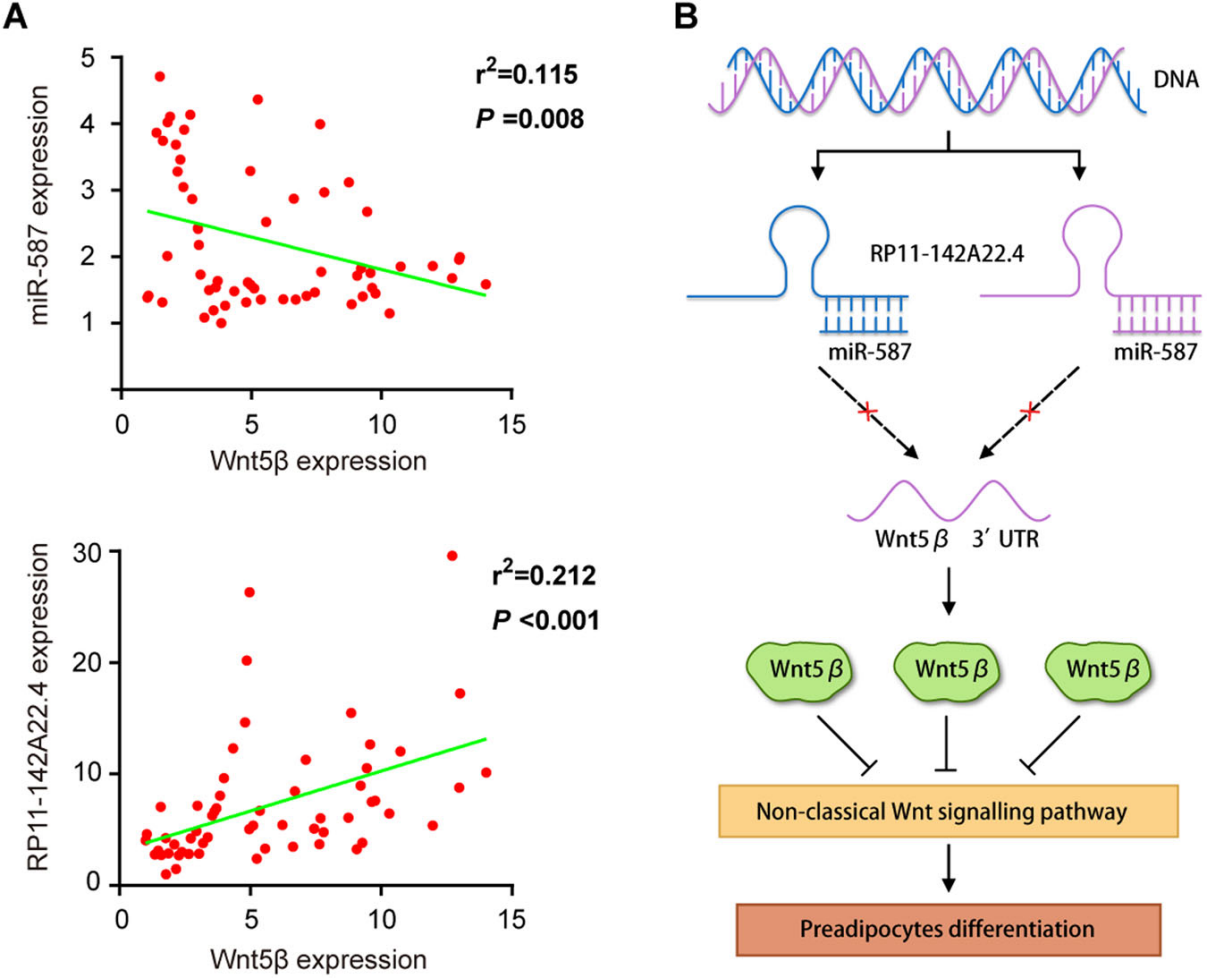
RP11-142A22.4 was positively correlated with Wnt5β expression. **a** Analysis of RP11-142A22.4 and miR-587/Wnt5β in adipose tissues, showing linear regressions and significant Pearson correlations. **b** RP11-142A22.4 might induce differentiation via the miR-587/Wnt5βsignaling pathway. Data are presented as means ± SD; significant difference was identified with Student’s *t* test. *P < 0.05; **P < 0.01; ns, not significant.

To explore the clinical implications of these findings, the expression of RP11-142A22.4, miR-587, and Wnt5β was analyzed in VAT from obese patients (*n* = 60) by qRT-PCR. Our results confirm that RP11-142A22.4 and Wnt5β expression is positively correlated, but miR-587 and Wnt5β expression is negatively correlated, in VAT (Fig. 6A). Collectively, these findings indicate that RP11-142A22.4 induces adipocyte differentiation via a miR-587/Wnt5β signaling pathway (Fig. 6b).

## Discussion

LncRNAs are emerging as an important new class of regulators that impact diverse biological processes and the pathogenesis of obesity^19,20^. Recent studies have confirmed the relationship between obesity and dysregulation of adipogenesis^21^. Some lncRNAs have been found to be associated with preadipocyte differentiation^9^ and adipogenesis^22^, and the altered expression of lncRNAs was correlated with the occurrence and development of obesity^18^. lncOb can control leptin expression by interacting with its proximal promoter. Additionally, mice with diet-induced obesity with lncOb knockout exhibit increased fat mass, with reduced plasma leptin levels^23^. Through interaction with hnRNPU and IGF2BP2 at distinct subcellular locations, linc-ADAL regulates adipocyte differentiation and lipogenesis^24^. Lnc-U90926 attenuates preadipocyte differentiation by inhibiting the transactivation of PPAR-γ^25^. In the present study, we found a novel regulator of preadipocyte differentiation, RP11-142A22.4, which is upregulated in the VAT of obese patients and predicts negative outcomes. Furthermore, RP11-142A22.4 promotes preadipocyte differentiation and acts as a ceRNA by sponging miR-587 to modulate Wnt5β expression.

LncRNAs have been reported to be located in both the nucleus and the cytoplasm^26,27^, and subcellular localization patterns of lncRNAs reveal fundamental insights into their biology and provide hypotheses for potential molecular roles. Recently, a novel mechanism of post-transcriptional regulation has been identified, i.e., that lncRNAs function as a natural miRNA sponge, interfere with miRNA pathways, and regulate the derepression of miRNA targets^28^. The miRNA sponge function of RP11-142A22.4 is consistent with the evidence from our study. First, using a coding potential calculator and a coding potential assessment tool, it is suggested that lncRNAs have very poor protein-coding potential. Second, RP11-142A22.4 was predominantly located in the cytoplasm and negatively correlated with miR-587 in the VAT of obese patients. Third, the interaction between RP11-142A22.4 and miR-587 was predicted using a bioinformatics database, which predicted that RP11-142A22.4 contains a miR-587 binding site. Fourth, miR-587 downregulation efficiently reversed the inhibition of differentiation by RP11-142A22.4 siRNA. Moreover, miR-587 upregulation significantly reduced differentiation in the presence of RP11-142A22.4. Finally, we found that RP11-142A22.4 could pull down miR-587; in fact, the highest binding affinity occurred between RP11-142A22.4 and miR-587. In addition to interaction with miRNAs, a single lncRNA could play additional roles in the cytoplasm, including in the regulation of different mechanisms.

A previous report demonstrated that the expression of PPAR-γ and C/EBP-α was increased in Wnt5β-overexpressing differentiated cells^17^. Wnt5β is able to promote adipogenesis in 3T3-L1 cells by inhibiting the canonical Wnt/β-catenin pathway^18,29^. In this study, using a bioinformatics database, miR-587 was predicted to target the 3′UTR of Wnt5β, which was confirmed using a dual-luciferase assay in preadipocytes. A previous study demonstrated that miR-587 could target the 3′UTR of PPP2R1B in colorectal cancer. Our data show that miR-587 targets Wnt5β more obviously than PPP2R1B^30^ in preadipocytes (Supplementary Fig. 11). Moreover, our findings indicate that lncRNAs can target miR-587 and subsequently promote Wnt5β and downstream PPAR-γ and C/EBP-α expression in preadipocytes. In addition, Wnt5β mRNA expression was relatively high in adipocytes obtained from obese patients who exhibited increased lncRNA expression. Therefore, these findings provide evidence for the notion that post-transcriptional regulation of Wnt5β by lncRNA depends on miR-587 in preadipocytes. However, RP11-142A22.4 is not conserved in mice, so the mechanisms underlying the regulation of differentiation in vivo still require further study. Moreover, in our study, both GO analysis and KEGG analysis also showed the important role of the differentially expressed lncRNAs identified through microarray analysis in the inflammatory response of adipocytes. Additionally, our lncRNA-miRNA-mRNA network indicated that lncRNAs may target inflammation-related miRNAs and mRNAs. However, further research is needed to understand the effect of lncRNAs on obesity-related inflammation.

In summary, we identified RP11-142A22.4 as an important factor associated with obesity, which plays a key role in adipocyte differentiation and adipogenesis. Moreover, we report the interaction between RP11-142A22.4 and miR-587 for the first time, and we reveal that RP11-142A22.4 regulates the expression of Wnt5β by sponging miR-587 in preadipocytes. Our results provide a foundation for further functional, diagnostic, and therapeutic studies related to RP11-142A22.4 in obese patients.

## Supplementary information

Supplemental figure 1

Supplemental figure 2

Supplemental figure 3

Supplemental figure 4

Supplemental figure 5

Supplemental figure 6

Supplemental figure 7

Supplemental figure 8

Supplemental figure 9

Supplemental figure 10

Supplemental figure 11

Supplemental figure legends

Table S4

Table S1

Table S2

Table S3

## Data Availability

LncRNA microarray datasets obtained from gene expression analyses in this study have been deposited to the NCBI GEO repository and are available through accession code GSE131819. Other relevant data supporting the findings of this study are available within the article and supplementary files or from the authors upon reasonable request.
