# Supplementary figures and images for: The lncRNA RP11-142A22.4 promotes adipogenesis by sponging miR-587 to modulate Wnt5β expression
Source: Cell Death Dis. 2020 Jun 19;11(6):475. doi: 10.1038/s41419-020-2550-9 (PMC7305230; doi:10.1038/s41419-020-2550-9)

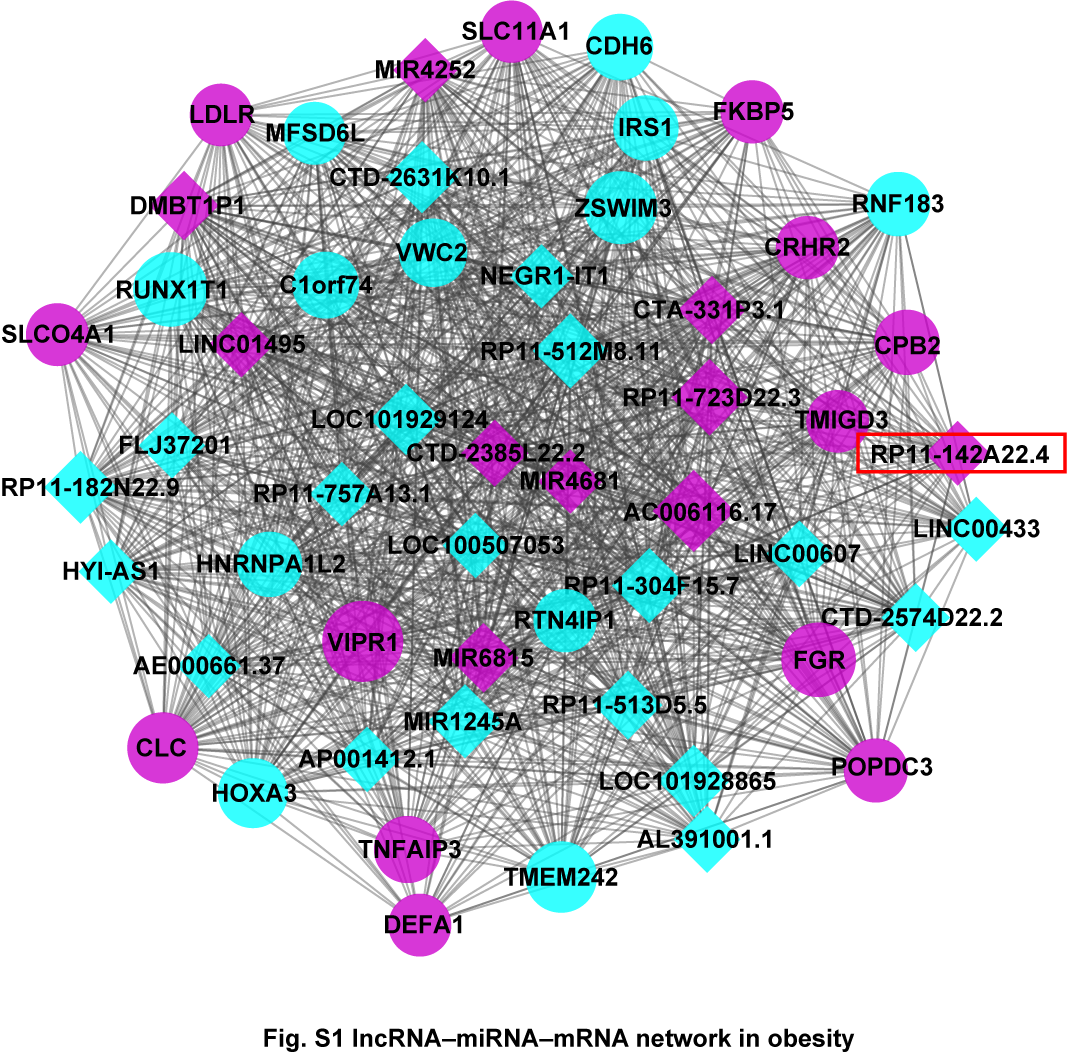

Supplement: Supplementary file 1 — Supplemental figure 1 [file 41419_2020_2550_MOESM1_ESM.tif]

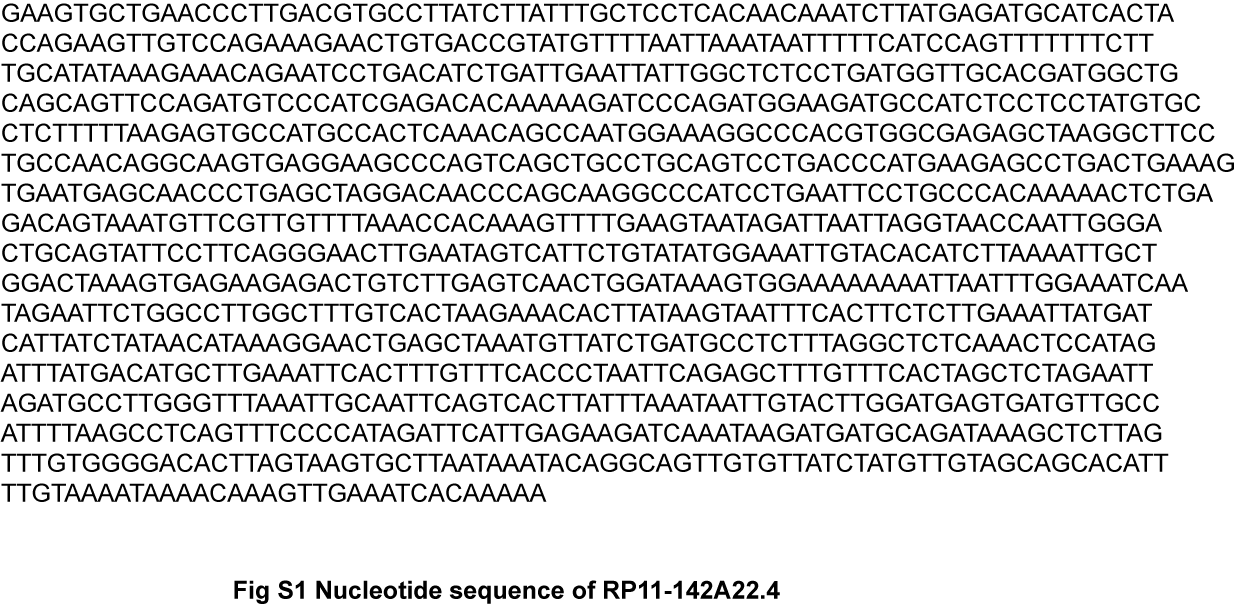

Supplement: Supplementary file 2 — Supplemental figure 2 [file 41419_2020_2550_MOESM2_ESM.tif]

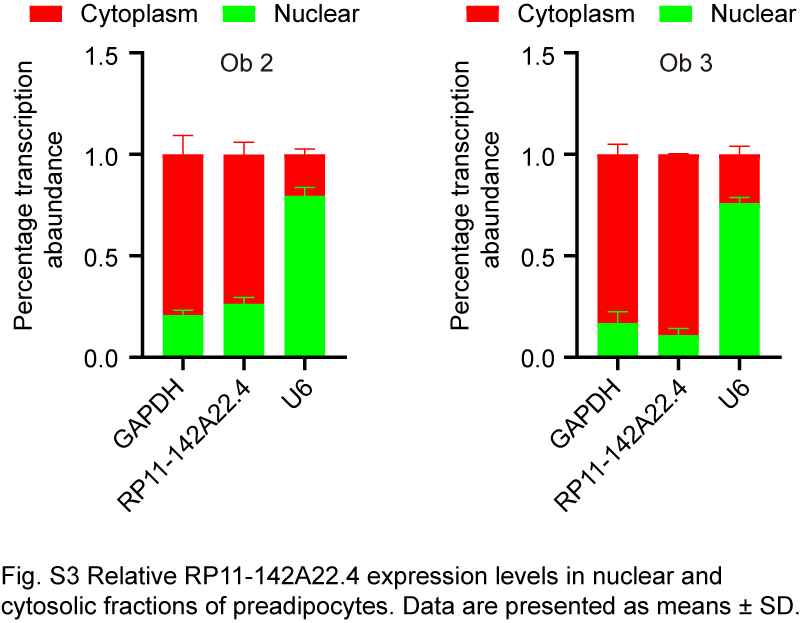

Supplement: Supplementary file 3 — Supplemental figure 3 [file 41419_2020_2550_MOESM3_ESM.tif]

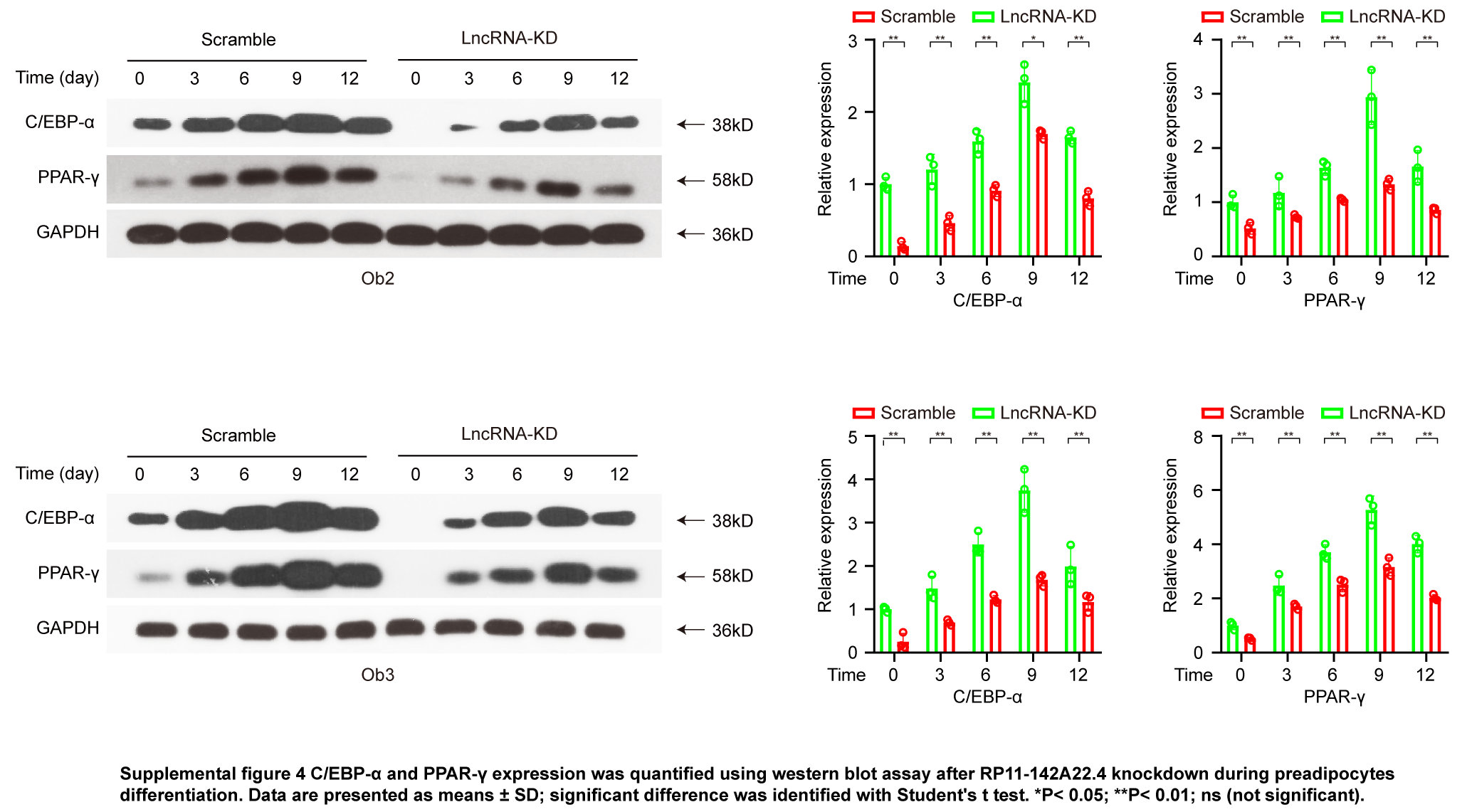

Supplement: Supplementary file 4 — Supplemental figure 4 [file 41419_2020_2550_MOESM4_ESM.tif]

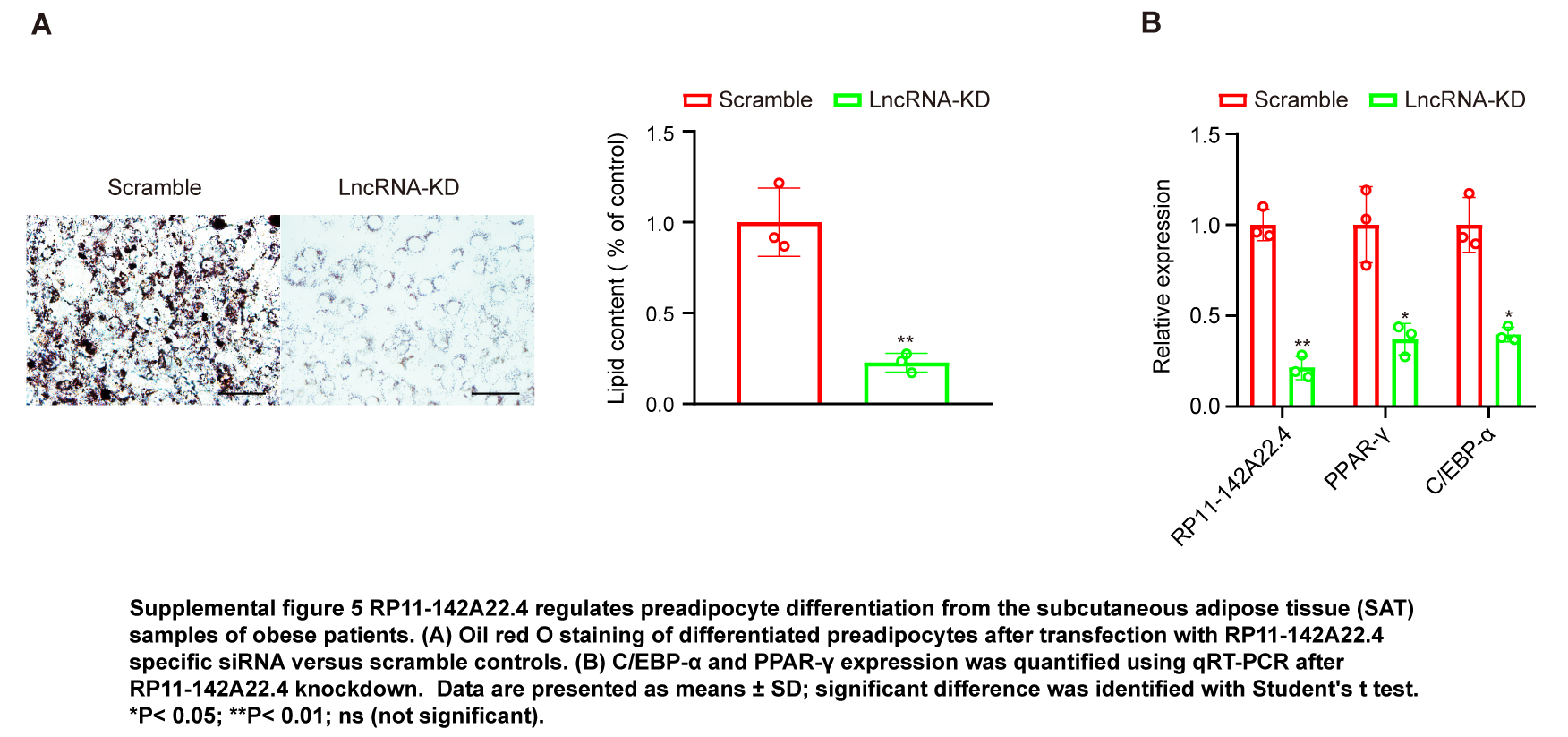

Supplement: Supplementary file 5 — Supplemental figure 5 [file 41419_2020_2550_MOESM5_ESM.tif]

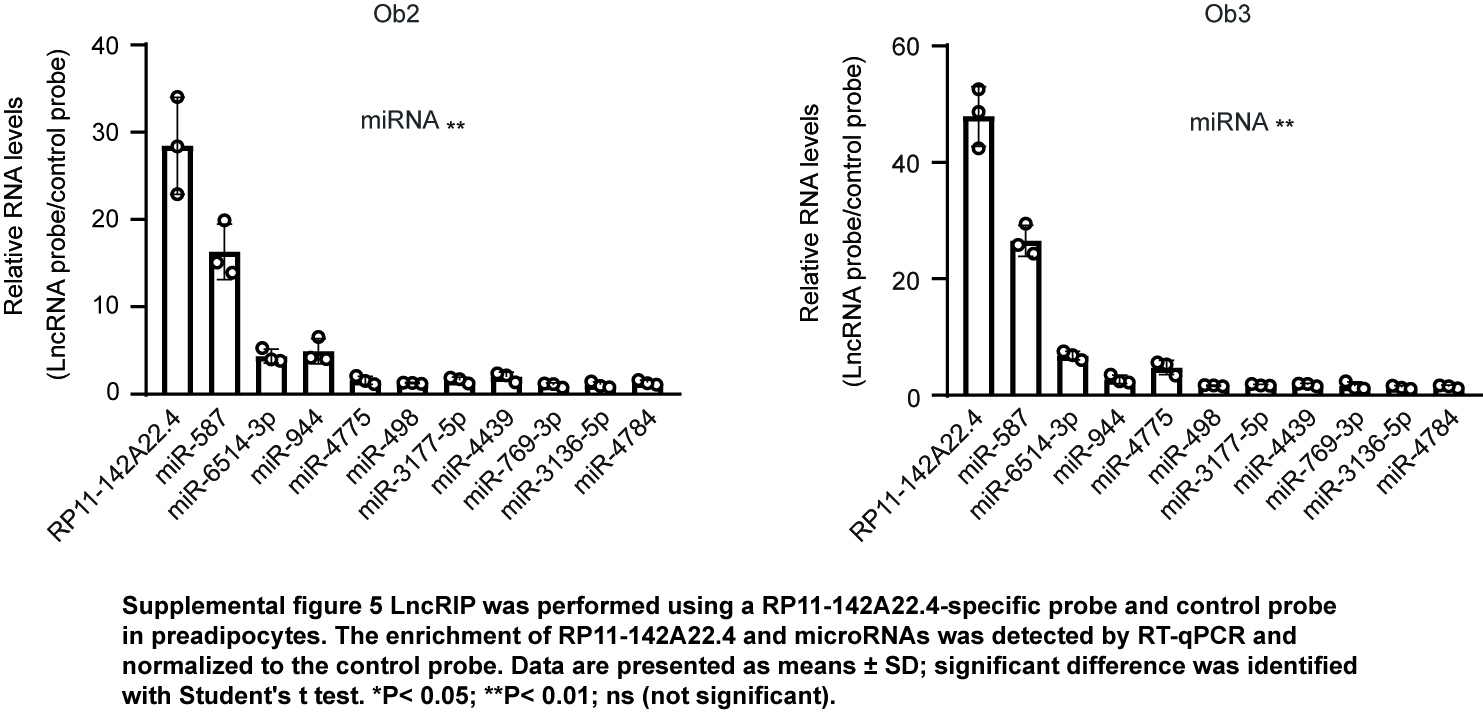

Supplement: Supplementary file 6 — Supplemental figure 6 [file 41419_2020_2550_MOESM6_ESM.tif]

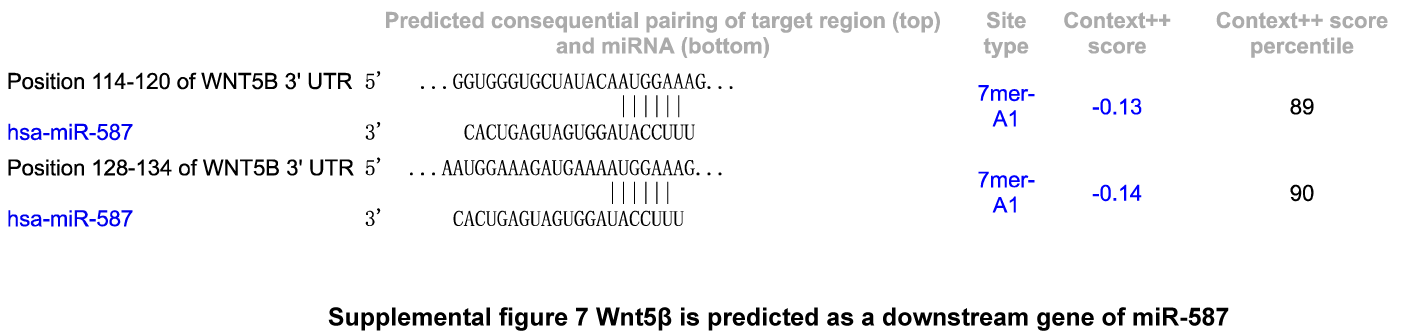

Supplement: Supplementary file 7 — Supplemental figure 7 [file 41419_2020_2550_MOESM7_ESM.tif]

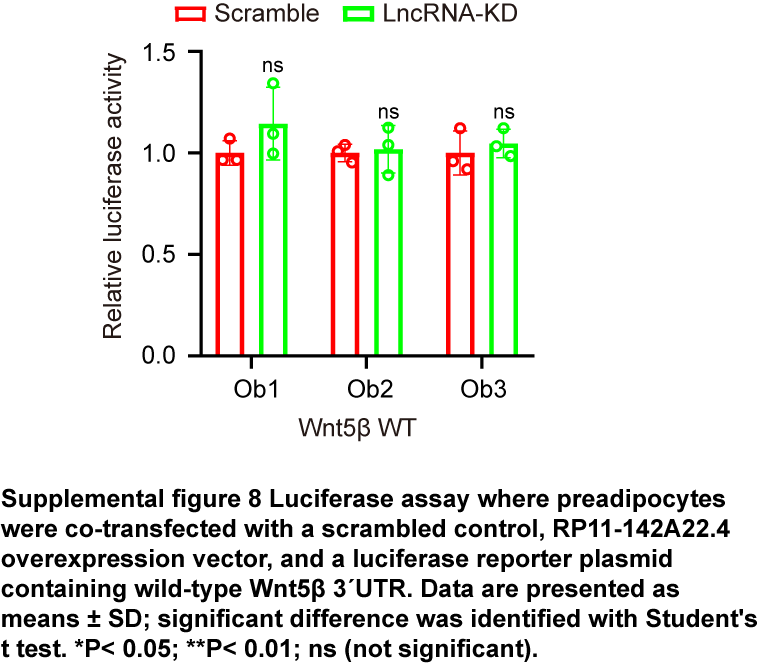

Supplement: Supplementary file 8 — Supplemental figure 8 [file 41419_2020_2550_MOESM8_ESM.tif]

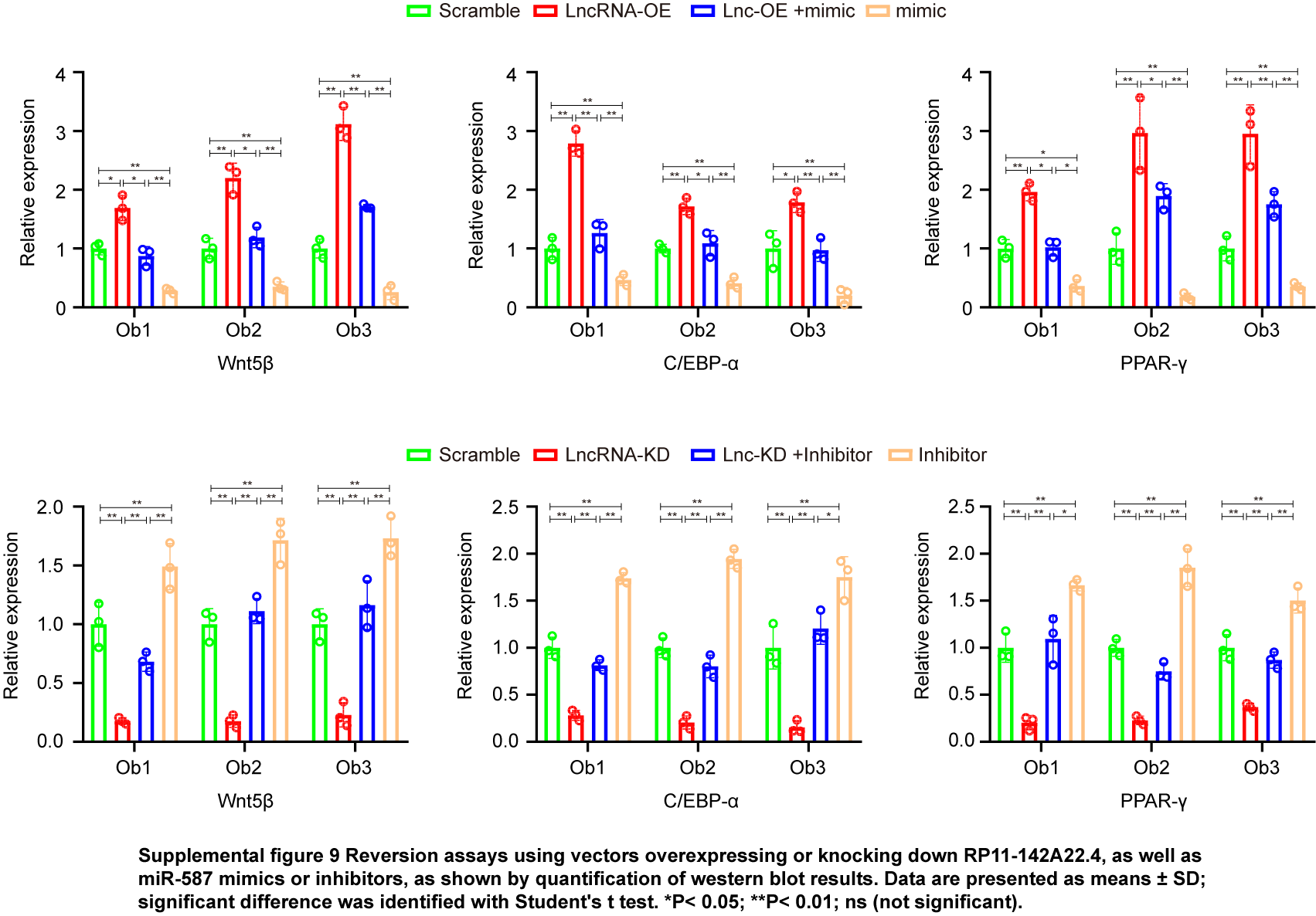

Supplement: Supplementary file 9 — Supplemental figure 9 [file 41419_2020_2550_MOESM9_ESM.tif]

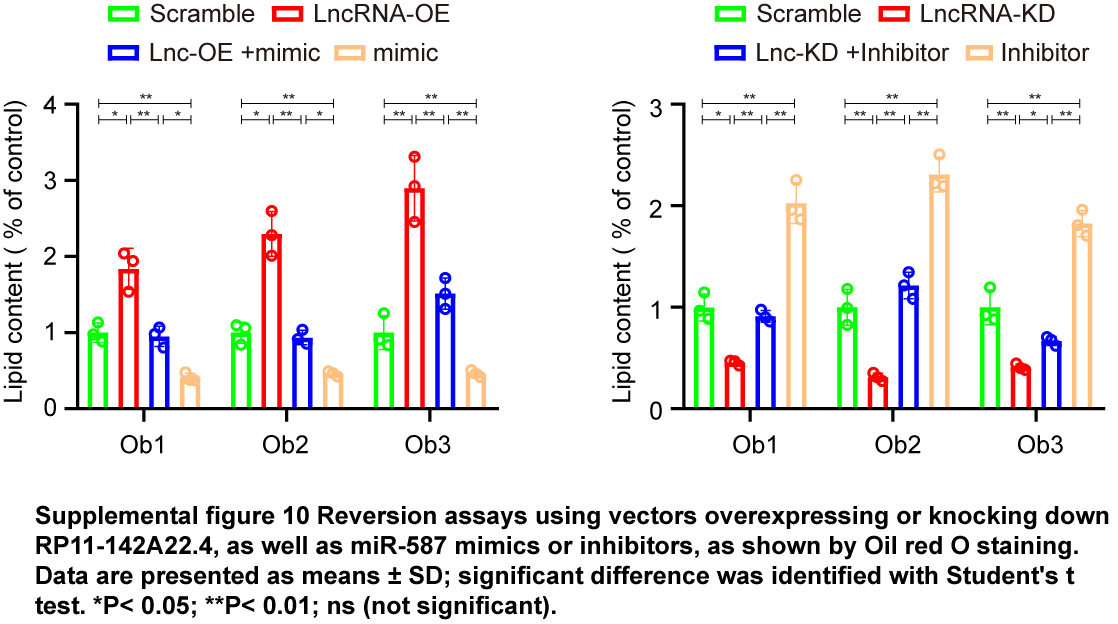

Supplement: Supplementary file 10 — Supplemental figure 10 [file 41419_2020_2550_MOESM10_ESM.tif]

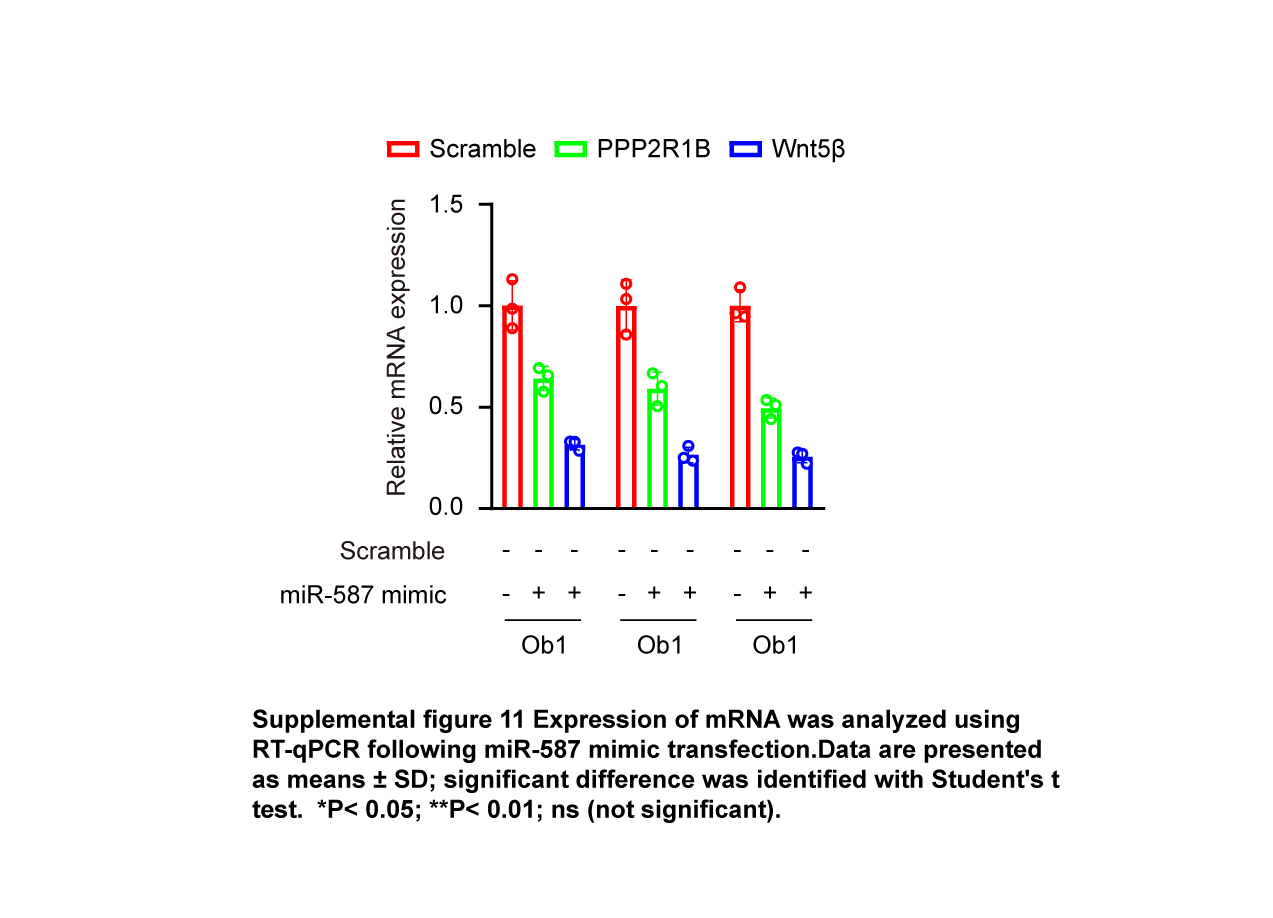

Supplement: Supplementary file 11 — Supplemental figure 11 [file 41419_2020_2550_MOESM11_ESM.tif]
