# Supplementary material for: The lncRNA RP11-142A22.4 promotes adipogenesis by sponging miR-587 to modulate Wnt5β expression
Source: Cell Death Dis. 2020 Jun 19;11(6):475. doi: 10.1038/s41419-020-2550-9 (PMC7305230; doi:10.1038/s41419-020-2550-9)
Supplement: Supplementary file 12 — Supplemental figure legends [file 41419_2020_2550_MOESM12_ESM.docx]

Supplemental figure 1 LncRNA–miRNA–mRNA network in obesity

Supplemental figure 2 Nucleotide sequence of RP11-142A22.4

Supplemental figure 3 Relative RP11-142A22.4 expression levels in nuclear and cytosolic fractions of preadipocytes. Data are presented as means ± SD; significant difference was identified with Student's t test. **P*< 0.05; ***P*< 0.01; ns (not significant).

Supplemental figure 4 C/EBP-α and PPAR-γ expression was quantified using western blot assay after RP11-142A22.4 knockdown during preadipocytes differentiation. Data are presented as means ± SD; significant difference was identified with Student's t test. **P*< 0.05; ***P*< 0.01; ns (not significant).

Supplemental figure 5 RP11-142A22.4 regulates preadipocyte differentiation from the subcutaneous adipose tissue (SAT) samples of obese patients. (**A**) Oil red O staining of differentiated preadipocytes after transfection with RP11-142A22.4 specific siRNA versus scramble controls. Scale bar = 100µm. **(B)** C/EBP-α and PPAR-γ expression was quantified using qRT-PCR after RP11-142A22.4 knockdown. Data are presented as means ± SD; significant difference was identified with Student's t test. **P*< 0.05; ***P*< 0.01; ns (not significant).

Supplemental figure 6 LncRIP was performed using a RP11-142A22.4-speciﬁc probe and control probe in preadipocytes. The enrichment of RP11-142A22.4 and microRNAs was detected by RT-qPCR and normalized to the control probe. Data are presented as means ± SD; significant difference was identified with Student's t test. **P*< 0.05; ***P*< 0.01; ns (not significant).

Supplemental figure 7 Wnt5β is predicted as a downstream gene of miR-587.

Supplemental figure 8 Luciferase assay where preadipocytes were co-transfected with a scrambled control, RP11-142A22.4 overexpression vector, and a luciferase reporter plasmid containing wild-type Wnt5β 3´UTR. Data are presented as means ± SD; significant difference was identified with Student's t test. *P< 0.05; **P< 0.01; ns (not significant).

Supplemental figure 9 Reversion assays using vectors overexpressing or knocking down RP11-142A22.4, as well as miR-587 mimics or inhibitors, as shown by quantification of western blot results. Data are presented as means ± SD; significant difference was identified with Student's t test. **P*< 0.05; ***P*< 0.01; ns (not significant).

Supplemental figure 10 Reversion assays using vectors overexpressing or knocking down RP11-142A22.4, as well as miR-587 mimics or inhibitors, as shown by Oil red O staining. Data are presented as means ± SD; significant difference was identified with Student's t test. **P*< 0.05; ***P*< 0.01; ns (not significant).

Supplemental figure 11 Expression of mRNA was analyzed using RT-qPCR following miR-587 mimic transfection. Data are presented as means ± SD; significant difference was identified with Student's t test. **P*< 0.05; ***P*< 0.01; ns (not significant).
