# Supplementary material for: The lncRNA RP11-142A22.4 promotes adipogenesis by sponging miR-587 to modulate Wnt5β expression
Source: Cell Death Dis. 2020 Jun 19;11(6):475. doi: 10.1038/s41419-020-2550-9 (PMC7305230; doi:10.1038/s41419-020-2550-9)
Supplement: Supplementary file 13 — Table S4 [file 41419_2020_2550_MOESM13_ESM.doc]

Table S4 miRNAs that might potentially bind to RP11-142A22.4

| miRNAs | Score |
| --- | --- |
| hsa-miR-587 | 0.953 |
| hsa-miR-6514-3p | 0.943 |
| hsa-miR-944 | 0.932 |
| hsa-miR-4775 | 0.923 |
| hsa-miR-498 | 0.914 |
| hsa-miR-3177-5p | 0.914 |
| hsa-miR-4439 | 0.913 |
| hsa-miR-769-3p | 0.901 |
| hsa-miR-3136-5p | 0.886 |
| hsa-miR-4784 | 0.882 |
| hsa-miR-3150b-3p | 0.878 |
| hsa-miR-3188 | 0.875 |
| hsa-miR-4687-3p | 0.872 |
| hsa-miR-3606-5p | 0.868 |
| hsa-miR-6721-5p | 0.865 |
| hsa-miR-1299 | 0.847 |
| hsa-miR-577 | 0.844 |
| hsa-miR-942-5p | 0.827 |
