# Supplementary material for: The lncRNA RP11-142A22.4 promotes adipogenesis by sponging miR-587 to modulate Wnt5β expression
Source: Cell Death Dis. 2020 Jun 19;11(6):475. doi: 10.1038/s41419-020-2550-9 (PMC7305230; doi:10.1038/s41419-020-2550-9)
Supplement: Supplementary file 14 — Table S1 [file 41419_2020_2550_MOESM14_ESM.docx]

| Table S1 The relationship between RP11-142A22.4 expression and clinic-pathological parameters in obese sample | | | | | | | | | | | | |
| --- | --- | --- | --- | --- | --- | --- | --- | --- | --- | --- | --- | --- |
| Name | Gender | Age (year) | Diabetes | Hyperuricemia | Hyperlipemia | Obstructive sleep apnea syndrome | Hyperinsulinemia (HINS) | Systolic BP (mm Hg) | Diastolic BP (mm Hg) | Preoperative weight(kg) | Height （cm） | RP11-142A22.4expression (qRT-PCR) |
| 1 | 0 | 30 | 0 | 0 | 1 | 0 | 0 | 111 | 76 | 72.0 | 155 | 1 |
| 2 | 0 | 32 | 0 | 0 | 1 | 1 | 0 | 134 | 76 | 91.0 | 155 | 1.496 |
| 3 | 0 | 30 | 0 | 0 | 0 | 0 | 0 | 112 | 69 | 84.0 | 164 | 2.418 |
| 4 | 0 | 37 | 0 | 0 | 1 | 1 | 1 | 130 | 82 | 70.0 | 156 | 2.716 |
| 5 | 0 | 28 | 0 | 0 | 1 | 1 | 0 | 110 | 78 | 74.0 | 157 | 2.734 |
| 6 | 0 | 44 | 0 | 0 | 1 | 1 | 0 | 138 | 90 | 73.0 | 158 | 2.791 |
| 7 | 0 | 26 | 0 | 0 | 0 | 0 | 0 | 124 | 74 | 79.0 | 159 | 2.848 |
| 8 | 0 | 39 | 0 | 0 | 1 | 0 | 0 | 115 | 75 | 71.0 | 147 | 2.862 |
| 9 | 0 | 26 | 0 | 0 | 0 | 1 | 0 | 121 | 70 | 120.0 | 168 | 2.875 |
| 10 | 0 | 43 | 0 | 0 | 1 | 1 | 0 | 125 | 84 | 84.0 | 157 | 3.018 |
| 11 | 1 | 33 | 1 | 0 | 1 | 1 | 0 | 160 | 105 | 130.0 | 188 | 3.096 |
| 12 | 0 | 19 | 1 | 0 | 0 | 0 | 0 | 155 | 114 | 103.0 | 164 | 3.142 |
| 13 | 1 | 30 | 1 | 0 | 1 | 1 | 0 | 146 | 80 | 123.0 | 179 | 3.255 |
| 14 | 1 | 28 | 0 | 0 | 0 | 0 | 0 | 182 | 90 | 103.0 | 165 | 3.316 |
| 15 | 0 | 33 | 0 | 0 | 1 | 0 | 0 | 100 | 70 | 92.0 | 162 | 3.482 |
| 16 | 0 | 31 | 0 | 1 | 1 | 0 | 1 | 128 | 74 | 69.0 | 153 | 3.688 |
| 17 | 1 | 23 | 0 | 1 | 1 | 1 | 0 | 125 | 85 | 121.0 | 175 | 3.709 |
| 18 | 1 | 28 | 0 | 1 | 0 | 1 | 0 | 142 | 105 | 100.0 | 167 | 3.803 |
| 19 | 0 | 31 | 0 | 0 | 0 | 0 | 0 | 152 | 106 | 75.5 | 153 | 3.84 |
| 20 | 0 | 28 | 0 | 0 | 0 | 1 | 0 | 136 | 96 | 95.0 | 161 | 4.067 |
| 21 | 1 | 31 | 0 | 0 | 1 | 1 | 0 | 146 | 86 | 120.0 | 174 | 4.234 |
| 22 | 0 | 35 | 0 | 0 | 0 | 1 | 0 | 144 | 87 | 94.0 | 160 | 4.263 |
| 23 | 0 | 28 | 0 | 0 | 1 | 0 | 0 | 112 | 63 | 75.0 | 158 | 4.348 |
| 24 | 0 | 21 | 0 | 0 | 0 | 0 | 0 | 120 | 78 | 105.0 | 168 | 4.617 |
| 25 | 0 | 33 | 0 | 0 | 1 | 1 | 0 | 116 | 74 | 100.0 | 162 | 4.791 |
| 26 | 1 | 34 | 0 | 0 | 1 | 1 | 1 | 134 | 88 | 97.3 | 167 | 4.875 |
| 27 | 0 | 28 | 0 | 0 | 1 | 1 | 0 | 125 | 94 | 114.0 | 178 | 4.876 |
| 28 | 0 | 27 | 0 | 0 | 0 | 0 | 0 | 124 | 88 | 118.5 | 162 | 5.053 |
| 29 | 0 | 35 | 1 | 0 | 1 | 0 | 1 | 152 | 105 | 80.5 | 165 | 5.117 |
| 30 | 0 | 37 | 1 | 1 | 1 | 0 | 1 | 140 | 74 | 117.0 | 164 | 5.377 |
| 31 | 0 | 29 | 1 | 0 | 0 | 1 | 1 | 120 | 70 | 96.5 | 160 | 5.387 |
| 32 | 1 | 28 | 0 | 0 | 1 | 1 | 0 | 129 | 80 | 129.0 | 172 | 5.429 |
| 33 | 0 | 23 | 0 | 0 | 1 | 1 | 0 | 131 | 85 | 104.0 | 162 | 6.043 |
| 34 | 0 | 26 | 1 | 0 | 1 | 1 | 0 | 129 | 83 | 95.0 | 163 | 6.089 |
| 35 | 1 | 19 | 0 | 1 | 0 | 1 | 0 | 131 | 79 | 130.0 | 171 | 6.289 |
| 36 | 0 | 27 | 0 | 0 | 0 | 1 | 0 | 126 | 74 | 108.0 | 163 | 6.464 |
| 37 | 0 | 34 | 0 | 0 | 1 | 0 | 1 | 116 | 79 | 100.0 | 163 | 6.667 |
| 38 | 0 | 24 | 0 | 0 | 0 | 0 | 0 | 102 | 63 | 110.0 | 170 | 6.707 |
| 39 | 0 | 31 | 0 | 1 | 0 | 1 | 0 | 127 | 95 | 89.0 | 154 | 6.928 |
| 40 | 0 | 29 | 0 | 0 | 0 | 0 | 0 | 120 | 70 | 89.0 | 160 | 7.074 |
| 41 | 1 | 26 | 0 | 1 | 0 | 1 | 0 | 136 | 72 | 139.0 | 169 | 7.164 |
| 42 | 0 | 24 | 0 | 0 | 0 | 0 | 0 | 126 | 85 | 123.0 | 164 | 7.518 |
| 43 | 0 | 24 | 0 | 0 | 1 | 1 | 0 | 120 | 74 | 111.0 | 165 | 7.601 |
| 44 | 0 | 23 | 0 | 1 | 1 | 1 | 0 | 117 | 80 | 87.0 | 162 | 8.067 |
| 45 | 0 | 28 | 0 | 0 | 0 | 1 | 0 | 139 | 84 | 110.0 | 160 | 8.46 |
| 46 | 1 | 22 | 0 | 0 | 1 | 1 | 0 | 135 | 70 | 120.0 | 180 | 8.798 |
| 47 | 0 | 24 | 1 | 0 | 0 | 1 | 0 | 129 | 76 | 101.0 | 174 | 8.965 |
| 48 | 1 | 36 | 0 | 0 | 1 | 1 | 0 | 128 | 74 | 141.5 | 180 | 9.631 |
| 49 | 0 | 22 | 0 | 0 | 1 | 0 | 0 | 140 | 67 | 105.0 | 163 | 10.143 |
| 50 | 0 | 20 | 0 | 0 | 0 | 0 | 0 | 127 | 81 | 93.0 | 159 | 10.525 |
| 51 | 0 | 22 | 0 | 0 | 1 | 1 | 0 | 112 | 68 | 118.5 | 162 | 11.294 |
| 52 | 0 | 22 | 0 | 1 | 1 | 1 | 0 | 112 | 74 | 80.0 | 162 | 12.025 |
| 53 | 1 | 25 | 0 | 1 | 1 | 1 | 0 | 120 | 78 | 135.0 | 193 | 12.317 |
| 54 | 0 | 24 | 0 | 0 | 1 | 1 | 0 | 118 | 89 | 72.5 | 155 | 12.67 |
| 55 | 0 | 22 | 0 | 0 | 0 | 0 | 0 | 140 | 80 | 152.0 | 176 | 14.642 |
| 56 | 0 | 24 | 1 | 1 | 1 | 1 | 0 | 122 | 74 | 123.0 | 161 | 15.484 |
| 57 | 0 | 23 | 0 | 0 | 1 | 1 | 0 | 136 | 86 | 115.0 | 160 | 17.245 |
| 58 | 0 | 24 | 0 | 1 | 0 | 1 | 0 | 150 | 86 | 150.0 | 162 | 20.182 |
| 59 | 1 | 25 | 0 | 0 | 1 | 1 | 0 | 151 | 91 | 139.0 | 166 | 26.303 |
| 60 | 0 | 24 | 0 | 0 | 0 | 1 | 0 | 138 | 76 | 145.0 | 172 | 29.59 |
|  |  |  |  |  |  |  |  |  |  |  |  |  |
