# Supplementary material for: The lncRNA RP11-142A22.4 promotes adipogenesis by sponging miR-587 to modulate Wnt5β expression
Source: Cell Death Dis. 2020 Jun 19;11(6):475. doi: 10.1038/s41419-020-2550-9 (PMC7305230; doi:10.1038/s41419-020-2550-9)
Supplement: Supplementary file 15 — Table S2 [file 41419_2020_2550_MOESM15_ESM.doc]

Table S2 Primers and probes used in the study

| Gene | | Sequence | Product length  （bp） |
| --- | --- | --- | --- |
| NONHSAT117148 | | 5’-CAGCGACTCCAGATCAGGTT-3’ | 112 |
|  |  | 3’-CCCCTTAGAATGGCTGTCCA-5’ |  |
| lnc-CYorf15A.1-2:6 | | 5’-TCTCAGTCACAGGACCGGAT-3’ | 106 |
|  |  | 3’-GGCTGGAGATGTAGCCATGT-5’ |  |
| PPP1R1C | | 5’-TGGGCTTGTTTTCCCGGTAG-3’ | 137 |
|  |  | 3’-TGAGCTGCCCATAGAGCTTG-5’ |  |
| CTD-2297D10.2 | | 5’-CGTCAGAATCATCACAACTGACA-3’ | 175 |
|  |  | 3’-CCCCTTTTATGGTGGGTGGT-5’ |  |
| lnc-BBS12-1:1 | | 5’-GTTGAGTGGCTCAGGGCTAA-3’ | 165 |
|  |  | 3’-GCAGGGGAACTTCTTCCACA-5’ |  |
| lnc-MAB21L3-4:1 | | 5’-TAGCCTGAAACACTGCCCTG-3’ | 100 |
|  |  | 3’-TTCTTGCCTCCATCAGGTGTC-5’ |  |
| RP11-142A22.4 | | 5’-TGAGAGCCTAAAGAGGCATCAG-3’ | 125 |
|  |  | 3’-TGAGAGCCTAAAGAGGCATCAG-5’ |  |
| RP11-12O16.1 | | 5’-CTGAGCTTCCCACTGGCTTT-3’ | 108 |
|  |  | 3’-AGTCTCTGCACAAACCGCAT-5’ |  |
| lnc-COL6A6-1:1 | | 5’-CTGTCCGTTCCACGGTTCC-3’ | 188 |
|  |  | 3’-AGTTTTTCTTGTCCCCAGACCT-5’ |  |
| PPAR-γ | | 5’-CGAGAGTCAGCCTTTAACGAAATG-3’ | 115 |
|  |  | 3’-AGGAGTGGGAGTGGTCTTCC-5’ |  |
| CEBP-α | | 5’-TATAGGCTGGGCTTCCCCTT-3’ | 94 |
|  |  | 3’-AGCTTTCTGGTGTGACTCGG-5’ |  |
| GAPDH | | 5’-GAAAGCCTGCCGGTGACTAA-3’ | 150 |
|  |  | 3’-GCCCAATACGACCAAATCAGAG-5’ |  |
|  |  |  |  |
| LncRNA probes | | | |
| RP11-142A22.4 | 5’- GGACTAAAGTGAGAAGAGACTGTCTTGAGTCAACTGGATA  -3’-biotin | |  |
| Control | 5’-CTAAGACTAGGTGTTCGGAGGGAAAACAAAAAGAGATATCAGAA -3’-biotin | |  |
| miRNA probes | | | |
| hsa-miR-587 | biotin-5’- CACTGAGTAGTGGATACCTTT -3’-biotin | |  |
| hsa-miR-6514-3p | biotin-5’- CTGCCTGTTCTTCCACTCCAG -3’-biotin | |  |
| hsa-miR-944 | biotin-5’- AAATTATTGTACATCGGATGAG -3’-biotin | |  |
| hsa-miR-4775 | biotin-5’- TTAATTTTTTGTTTCGGTCACT -3’-biotin | |  |
| hsa-miR-498 | biotin-5’- TTTCAAGCCAGGGGGCGTTTTTC -3’-biotin | |  |
| hsa-miR-3177-5p | biotin-5’- TGTGTACACACGTGCCAGGCGCT -3’-biotin | |  |
| hsa-miR-4439 | biotin-5’- GTGACTGATACCTTGGAGGCAT -3’-biotin | |  |
| hsa-miR-769-3p | biotin-5’- CTGGGATCTCCGGGGTCTTGGTT -3’-biotin | |  |
| hsa-miR-3136-5p | biotin-5’- CTGACTGAATAGGTAGGGTCATT -3’-biotin | |  |
| hsa-miR-4784 | biotin-5’- TGAGGAGATGCTGGGACTGA -3’-biotin | |  |
| FISH probes | | | |
| RP11-142A22.4 | digoxin-5’-GGACTAAAGTGAGAAGAGACTGTCTTGAGTCAACTGGATAAAGT -3’-digoxin | |  |
| Negative Control | digoxin-5’-TAAGACTAGGTGTTCGGAGGGAAAACAAAAAGAGATATCAGAA -3’- digoxin | |  |
| GAPDH | digoxin-5’-AGGCGCCCAATACGACCAAATCAGAGAATAATCTAGGAAAAGCA -3’-digoxin | |  |
| siRNAs |  | |  |
| RP11-142A22.4 | 5’-AGCACAAGUACAAGAAUCAUU -3’ | |  |
| Wnt5β | 5’-ACAUACUCUUUACUUCAUCAG -3’ | |  |
