# Supplementary material for: The lncRNA RP11-142A22.4 promotes adipogenesis by sponging miR-587 to modulate Wnt5β expression
Source: Cell Death Dis. 2020 Jun 19;11(6):475. doi: 10.1038/s41419-020-2550-9 (PMC7305230; doi:10.1038/s41419-020-2550-9)
Supplement: Supplementary file 16 — Table S3 [file 41419_2020_2550_MOESM16_ESM.docx]

| Table S3 Differential expression lncRNAs between the obese and lean VAT samples using microarray detection | | | | | | | | | |
| --- | --- | --- | --- | --- | --- | --- | --- | --- | --- |
| LncRNA ID | pvalues | foldchange | Obese 1 | Obese2 | Obese 3 | Lean 1 | Lean 2 | Lean 3 | GeneSymbol |
| NONHSAT117148 | 0.024138732 | 15.7345554 | 4.680575975 | 3.130386774 | 5.0882102 | 0.554361277 | 0.507454131 | 0.54424412 | --- |
| lnc-CYorf15A.1-2:6 | 0.031341216 | 12.66229839 | 6.187187772 | 3.780950571 | 6.419438 | 0.754938797 | 1.725684645 | 3.105181023 | --- |
| NR_048567 | 0.00278068 | 0.129270984 | 0.54774561 | 0.541890126 | 0.5476109 | 3.78486025 | 3.378612115 | 3.276956961 | PPP1R1C |
| lnc-ITGAX-2:2 | 0.037580388 | 7.276363978 | 4.596093258 | 3.820599368 | 5.4596446 | 0.605715881 | 0.629939099 | 3.043399629 | --- |
| NR_109915 | 0.015089301 | 0.13809275 | 4.640402351 | 5.59814526 | 3.9297869 | 7.220802542 | 8.539113565 | 6.913980157 | CTD-2297D10.2 |
| lnc-BBS12-1:1 | 0.00215353 | 6.921110381 | 4.668229774 | 5.234664131 | 4.2385607 | 1.508156202 | 1.855890016 | 2.426836321 | --- |
| lnc-MAB21L3-4:1 | 0.024263042 | 5.974278311 | 3.797975722 | 5.374715397 | 4.3853738 | 1.109527722 | 1.434754949 | 2.983706694 | --- |
| ENST00000610239 | 0.001088032 | 5.609402705 | 3.176769255 | 3.439629254 | 3.7950933 | 1.409244162 | 0.756953813 | 0.744271148 | RP11-142A22.4 |
| ENST00000623516 | 0.040474339 | 5.416153532 | 6.274521224 | 8.145640946 | 6.6570384 | 5.234217465 | 4.276968471 | 4.816891208 | RP11-12O16.1 |
| lnc-COL6A6-1:1 | 0.036500753 | 0.188516196 | 0.987862912 | 2.522988611 | 0.9620211 | 3.208426304 | 4.981989662 | 3.391855167 | --- |
| NR_003347 | 0.014524362 | 0.18948694 | 1.246450585 | 2.10520116 | 0.5491816 | 4.323474796 | 3.593557896 | 3.440489159 | SNORD115-32 |
| ENST00000621884 | 0.014039689 | 5.080767075 | 3.053621829 | 4.421948411 | 3.7446661 | 1.066330031 | 1.793273967 | 1.551593725 | LA16c-329F2.2 |
| NONHSAT118504 | 0.038994928 | 5.073482405 | 9.792502648 | 8.554241011 | 9.9145516 | 8.100073578 | 6.731059587 | 5.870276593 | --- |
| ENST00000614781 | 0.006110606 | 5.030465202 | 7.636102143 | 8.76768823 | 8.1715821 | 6.131548627 | 6.16286793 | 5.383532656 | CTD-3128G10.7 |
| lnc-ABCB5-4:1 | 0.037329092 | 0.211458227 | 4.542009117 | 4.110762637 | 4.6209395 | 6.403675269 | 7.403619755 | 5.761539827 | --- |
| ENST00000582263 | 0.003717252 | 0.217155027 | 4.922220582 | 5.833021426 | 5.5820712 | 7.217926687 | 7.76766745 | 7.999657006 | RP11-387H17.4 |
| lnc-MAB21L3-3:1 | 0.000725908 | 4.498256559 | 7.32981806 | 7.8841446 | 7.7044383 | 5.175667284 | 5.488056953 | 5.744886298 | --- |
| NR_120420 | 0.047216104 | 4.445336586 | 4.187946384 | 3.041140377 | 3.4259367 | 0.492963964 | 2.464640127 | 0.528341714 | LOC100507006 |
| NR_031647 | 0.021904679 | 0.225105228 | 3.687440478 | 3.48257909 | 3.2910285 | 5.479574563 | 6.221257678 | 4.960922543 | MIR1245A |
| ENST00000605233 | 0.01884503 | 0.227186625 | 1.706407367 | 0.534056289 | 0.5855285 | 3.181565348 | 2.937874978 | 3.407036091 | RP11-734K2.4 |
| lnc-RUNX1T1-6:1 | 0.035523337 | 0.229488906 | 9.824177905 | 9.575122471 | 9.9147849 | 11.63000521 | 12.56826165 | 11.12760247 | --- |
| lnc-HEBP1-2:1 | 0.036781179 | 0.236901032 | 9.981335059 | 9.742503448 | 9.976794 | 11.75034773 | 12.62330105 | 11.2178023 | --- |
| lnc-TSPAN9-2:1 | 0.039157186 | 0.238443978 | 10.35631343 | 10.24994593 | 10.312743 | 12.18638246 | 12.99446686 | 11.60596568 | --- |
| lnc-AF165138.7.1-6:1 | 0.01761889 | 4.191061522 | 6.946318747 | 5.882502903 | 7.1611945 | 4.202483721 | 4.617139834 | 5.116916697 | --- |
| lnc-FBP2-3:1 | 0.033386296 | 0.241177337 | 2.673733647 | 0.960752222 | 1.9820109 | 3.647437831 | 4.340047732 | 4.179737946 | --- |
| lnc-CCDC90B-4:1 | 0.010956775 | 0.242028414 | 3.177836648 | 2.575618078 | 2.8668688 | 5.277531004 | 5.105434852 | 4.24389017 | --- |
| lnc-TMC3-1:1 | 0.048027764 | 0.242716837 | 2.593688648 | 1.960511092 | 1.2829923 | 3.190787194 | 3.504833667 | 4.928290408 | --- |
| ENST00000610240 | 0.03825772 | 4.078685148 | 4.881828824 | 4.963718663 | 6.1003911 | 2.727184341 | 2.551751369 | 4.264273523 | RP11-302L19.3 |
| lnc-NDFIP1-8:1 | 0.03598152 | 4.071666796 | 3.269915987 | 4.055967246 | 3.9130357 | 2.60226263 | 0.984914652 | 1.071903134 | --- |
| ENST00000603682 | 0.030247477 | 0.24689928 | 3.882164136 | 2.350762974 | 2.4922398 | 4.627604633 | 5.749436655 | 4.623719857 | RP1-142L7.9 |
| lnc-DDX58-6:2 | 0.017285343 | 0.248337787 | 11.57831697 | 11.32817655 | 11.610368 | 13.27843715 | 14.04700203 | 13.03517876 | --- |
| lnc-ABL2-2:1 | 0.043122348 | 0.251210698 | 11.19758155 | 10.76075034 | 11.071896 | 12.84912648 | 13.66429011 | 12.12403383 | --- |
| lnc-SOCS4-6:1 | 0.032616471 | 3.910736163 | 3.311396078 | 4.544069122 | 3.7914848 | 2.117278721 | 2.541435486 | 0.913953981 | --- |
| lnc-UPB1-1:1 | 0.032950538 | 3.906928676 | 3.024100807 | 3.750868843 | 3.0080227 | 2.140998696 | 0.595984479 | 0.735359847 | --- |
| lnc-HSPA8-1:1 | 0.020525606 | 0.256063008 | 11.39179361 | 11.23830408 | 11.399034 | 13.04706458 | 13.82831866 | 12.86593352 | --- |
| NR_024376 | 0.031502741 | 0.256228964 | 4.154198466 | 2.844766933 | 4.352553 | 5.4274776 | 6.206698352 | 5.915518829 | FAM225B |
| lnc-PLXNA2-5:1 | 0.02104738 | 0.257400469 | 11.89369965 | 11.7341791 | 11.837138 | 13.62969686 | 14.26894903 | 13.2608473 | --- |
| lnc-C9orf3-2:1 | 0.02039563 | 0.258158158 | 11.89099507 | 11.63942402 | 11.827951 | 13.58388966 | 14.24958764 | 13.19758602 | --- |
| lnc-BTF3L4-5:1 | 0.023761828 | 0.259461184 | 11.54411902 | 11.29845721 | 11.382528 | 13.19908869 | 13.88169425 | 12.77487242 | --- |
| lnc-SCGB2B2-6:1 | 0.01602689 | 0.26129777 | 12.1355328 | 11.87386031 | 12.015761 | 13.78901156 | 14.42099634 | 13.46913766 | --- |
| lnc-CHMP4C-8:1 | 0.012259481 | 0.261323443 | 11.77727883 | 11.4766017 | 11.750348 | 13.37910967 | 14.08865171 | 13.20516033 | --- |
| lnc-VAMP3-3:1 | 0.00265573 | 3.814780115 | 4.783393383 | 4.262200107 | 4.9026095 | 2.714282255 | 2.544742381 | 2.94340736 | --- |
| lnc-TLK1-5:1 | 0.010800572 | 0.26391463 | 12.37115503 | 12.0848437 | 12.275569 | 13.963524 | 14.61649632 | 13.7962989 | --- |
| lnc-OCRL-1:1 | 0.012636617 | 0.265024241 | 12.06242472 | 11.78473626 | 12.048703 | 13.70261751 | 14.34944243 | 13.45518921 | --- |
| NR_001276 | 0.004316703 | 3.770814366 | 4.350763995 | 3.911555348 | 4.1870373 | 1.999266845 | 2.664738336 | 1.960936861 | SNORD56B |
| lnc-NDEL1-6:1 | 0.023806797 | 0.266020728 | 11.95620282 | 11.82295635 | 11.940903 | 13.65427451 | 14.31880208 | 13.28870618 | --- |
| lnc-FAM49A-9:1 | 0.029455247 | 0.26604673 | 11.3179021 | 11.17320463 | 11.20155 | 12.87842557 | 13.70743493 | 12.60288562 | --- |
| lnc-PAPSS2-3:1 | 0.017552114 | 0.270109401 | 11.97087178 | 11.75774955 | 11.876924 | 13.63429238 | 14.21310962 | 13.2729309 | --- |
| lnc-SLAIN1-6:1 | 0.024438635 | 3.683898955 | 4.687280959 | 4.244622913 | 5.5919588 | 2.84956047 | 2.510358505 | 3.623753043 | --- |
| lnc-CRTAM-2:1 | 0.045650977 | 3.654704243 | 2.780314696 | 3.757137301 | 4.4699517 | 1.625648923 | 1.434679997 | 2.549440599 | --- |
| ENST00000509144 | 0.048596891 | 0.274313508 | 2.325584601 | 0.652638233 | 2.1094448 | 3.697044283 | 4.041575343 | 3.3643279 | ZFPM2-AS1 |
| lnc-CERS5-2:2 | 0.038536273 | 0.274901949 | 7.438438227 | 6.588183176 | 7.1220131 | 8.763168242 | 9.616507553 | 8.067293147 | --- |
| lnc-CXCR1-1:1 | 0.033677443 | 3.606913155 | 4.754997095 | 4.405893908 | 3.8188431 | 1.549757654 | 2.390456964 | 3.186650707 | --- |
| lnc-BAIAP2L1.1-3:1 | 0.033868245 | 0.277592936 | 11.12284467 | 10.93539009 | 11.098206 | 12.75747469 | 13.4515179 | 12.24633993 | --- |
| lnc-NOX3-7:1 | 0.002097949 | 3.599372016 | 3.956374015 | 3.587063058 | 4.1863133 | 2.356072456 | 1.700338652 | 2.117717979 | --- |
| lnc-PARN-12:1 | 0.037167884 | 0.280214614 | 11.5803004 | 11.40154733 | 11.574211 | 13.18276005 | 13.92726387 | 12.68533945 | --- |
| NR_004384 | 0.008988083 | 3.568107949 | 9.898902877 | 10.81380915 | 10.015968 | 8.901782739 | 8.156782324 | 8.221744566 | SNAR-F |
| ENST00000616576 | 0.042800619 | 3.554660238 | 5.689029795 | 4.087407113 | 4.8106237 | 3.167478754 | 2.606227427 | 3.598927282 | RP11-87C12.5 |
| NONHSAT127683 | 0.018666314 | 0.282469561 | 6.102173886 | 5.272810657 | 5.0750074 | 7.343442073 | 7.878406628 | 6.668030741 | --- |
| lnc-ASB1-4:1 | 0.049779485 | 0.28391107 | 4.322235196 | 3.570825057 | 2.7847538 | 4.819164833 | 6.27492377 | 4.950509007 | --- |
| lnc-GJA8-3:1 | 0.029087648 | 0.289863434 | 1.574236579 | 2.082585577 | 0.6270735 | 2.857242212 | 3.330931932 | 3.68984333 | --- |
| lnc-FAM55C-2:1 | 0.049544731 | 3.414485386 | 3.1452265 | 4.655475292 | 4.3633129 | 2.245669744 | 2.071590615 | 2.818405989 | --- |
| ENST00000541707 | 0.040322791 | 0.293362208 | 1.782630917 | 2.61092766 | 3.1924835 | 4.707764778 | 4.738564536 | 3.444345932 | RP11-81H14.2 |
| lnc-CHI3L1-2:3 | 0.038407967 | 3.397843014 | 4.834785589 | 3.618128974 | 3.4769807 | 2.427789999 | 2.752671983 | 1.664084515 | --- |
| ENST00000459965 | 0.01326429 | 3.368844933 | 4.959491922 | 5.681858733 | 5.8047871 | 3.806837291 | 3.123126547 | 4.198452332 | RP11-723D22.3 |
| lnc-TAS2R50-1:1 | 0.007785684 | 0.297964004 | 3.830006018 | 3.038573647 | 3.6423802 | 5.425356466 | 4.738151963 | 5.571447027 | --- |
| ENST00000622079 | 0.007359163 | 0.298526439 | 3.485009716 | 3.552781288 | 2.7460906 | 4.846699512 | 5.460650545 | 4.726819836 | LINC00869 |
| NR_105045 | 0.00500847 | 0.301912465 | 1.643457566 | 0.981908006 | 1.6833044 | 3.329572397 | 2.890181391 | 3.330637512 | LOC101927934 |
| lnc-CNPY2-2:1 | 0.041405263 | 0.311240528 | 2.504474237 | 0.961446844 | 1.7671734 | 3.900817121 | 3.395488048 | 3.316789919 | --- |
| NONHSAT017417 | 0.049366981 | 3.206642867 | 3.400955908 | 3.779692686 | 3.1077942 | 2.563472484 | 0.982036073 | 1.250336135 | --- |
| NR_026703 | 0.006409179 | 3.199155655 | 7.90336585 | 8.391350579 | 8.2535022 | 6.663873482 | 6.769790352 | 6.014990721 | VTRNA1-1 |
| ENST00000410230 | 0.045143766 | 0.314736529 | 3.088506296 | 3.219192257 | 4.2061598 | 5.626730372 | 5.538040177 | 4.276968471 | RN7SKP151 |
| lnc-FOXD4L2-1:3 | 0.013414782 | 0.315828016 | 3.518642659 | 3.571669152 | 2.830246 | 4.601198763 | 5.52665357 | 4.705053519 | --- |
| lnc-UPK1B-2:1 | 0.040960732 | 0.315940386 | 11.38252778 | 11.28964321 | 11.360119 | 12.85477994 | 13.54124217 | 12.38943981 | --- |
| lnc-CMPK2-29:1 | 0.042904315 | 0.316912823 | 1.73343485 | 2.66701691 | 2.4197471 | 3.464752088 | 3.396325489 | 4.691941618 | --- |
| NR_024342 | 0.015959243 | 3.146250288 | 10.6752804 | 11.67394822 | 11.400906 | 10.06994035 | 9.094770429 | 9.63677881 | SNAR-H |
| lnc-C9orf156-2:1 | 0.034719573 | 0.318070557 | 3.086137688 | 1.636457423 | 2.107298 | 3.609745797 | 4.533902249 | 3.872546101 | --- |
| ENST00000622358 | 0.042495387 | 0.319022141 | 2.278616321 | 1.205525557 | 0.7037062 | 2.559298435 | 3.492028617 | 3.378237778 | KCNQ1OT1_1 |
| NR_036581 | 0.005909639 | 0.319615338 | 3.492073666 | 3.494090025 | 3.4635479 | 5.352475706 | 5.090305778 | 4.909234193 | LINC00675 |
| ENST00000560727 | 0.034355472 | 0.321633839 | 3.693544695 | 2.838684128 | 3.7885146 | 4.610557746 | 4.670841626 | 5.795169424 | RP11-707P17.2 |
| lnc-TRMT11-4:1 | 0.046290702 | 0.323214182 | 3.946162527 | 3.950514139 | 2.8275151 | 5.549896358 | 5.651607098 | 4.348274367 | --- |
| lnc-IL15RA-4:1 | 0.047648045 | 3.075771434 | 3.559795806 | 4.311007342 | 5.0465484 | 2.130396989 | 3.232496475 | 2.859001554 | --- |
| ENST00000616370 | 0.012602444 | 0.325386606 | 3.378918958 | 2.439133498 | 3.1912649 | 4.642634447 | 4.346339979 | 4.972199113 | RP11-287D1.4 |
| lnc-AL669831.1-3:2 | 0.046336673 | 3.068492754 | 3.121907569 | 3.04913325 | 3.4355002 | 1.132365312 | 0.944299913 | 2.305649238 | --- |
| lnc-AKR1D1-5:1 | 0.018187341 | 3.054301479 | 4.54794386 | 3.473900242 | 4.1639613 | 2.281494272 | 2.221912439 | 2.933304175 | --- |
| lnc-SUN3-4:1 | 0.021130099 | 3.041417866 | 7.228342736 | 7.120529815 | 7.6168239 | 5.681238256 | 5.094670587 | 6.211613948 | --- |
| NR_024243 | 0.035139019 | 3.027699588 | 11.36934064 | 12.39459691 | 12.385364 | 11.03138509 | 9.708681112 | 10.54056258 | SNAR-D |
| NONHSAT128718 | 0.045461045 | 3.011576331 | 4.048963086 | 3.033221446 | 4.1321835 | 2.51474528 | 1.24991952 | 2.571407201 | --- |
| lnc-RP3-377D14.1.1-6:2 | 0.017366451 | 0.33225162 | 7.078319846 | 6.042156499 | 6.4053998 | 7.798106045 | 8.648314036 | 7.885063035 | --- |
| ENST00000504210 | 0.022868032 | 0.333319996 | 3.616519294 | 2.596399431 | 3.2261066 | 4.212083517 | 5.308694012 | 4.634963446 | RP11-834C11.8 |
| lnc-EHF-7:1 | 0.003608925 | 0.333847973 | 7.731280483 | 7.745772536 | 7.6963715 | 9.395437167 | 9.409611567 | 9.095796251 | --- |
| lnc-DHX34-1:1 | 0.031829424 | 2.970304266 | 4.788074599 | 3.945146748 | 3.5548531 | 2.970074642 | 2.668050928 | 2.088234712 | --- |
| ENST00000555776 | 0.039420806 | 2.965191279 | 3.451189919 | 3.966114489 | 4.7741362 | 2.942786577 | 2.754635204 | 1.906676975 | RP11-61O1.1 |
| NONHSAT140083 | 0.036640299 | 2.964721919 | 4.18681209 | 4.180579406 | 5.3200103 | 2.552209247 | 3.328680464 | 3.295886988 | --- |
| lnc-PRH1-1:9 | 0.015310464 | 0.337354638 | 4.097533173 | 3.503564572 | 3.3033401 | 5.225416291 | 4.669764307 | 5.665700091 | --- |
| ENST00000564549 | 0.003969525 | 2.955715083 | 5.191857129 | 5.083913929 | 5.7148173 | 3.651933659 | 3.579170545 | 4.094943917 | RP11-209D14.2 |
| NR_024244 | 0.01535797 | 2.949075878 | 10.60373235 | 11.48825339 | 11.209858 | 10.00268379 | 9.024058567 | 9.566962405 | SNAR-G2 |
| ENST00000561181 | 0.037427264 | 2.943720492 | 2.89770139 | 4.198592198 | 3.4736977 | 2.366726773 | 1.381916801 | 2.256742209 | CTD-3065J16.6 |
| ENST00000561663 | 0.014201731 | 2.902330864 | 3.743106083 | 3.813829351 | 3.7907645 | 2.427700383 | 1.834163388 | 2.400966333 | RP11-343H19.1 |
| lnc-NPFFR1-1:1 | 0.000450559 | 2.901768739 | 5.724164745 | 6.041876753 | 5.7582937 | 4.509009304 | 4.234979568 | 4.168020724 | --- |
| lnc-GLRX-3:1 | 0.013129487 | 2.89062236 | 5.810348669 | 6.72280873 | 6.3402734 | 4.762519028 | 4.359649542 | 5.182322087 | --- |
| ENST00000601096 | 0.042960183 | 2.88501059 | 4.438296992 | 3.324891535 | 4.5750341 | 2.406330431 | 2.305719077 | 3.170641611 | RP11-381O7.3 |
| NR_027046 | 0.001239168 | 2.877839806 | 7.178048085 | 7.158294272 | 7.571781 | 5.848575065 | 5.960799835 | 5.528515303 | LOC145474 |
| NR_024220 | 0.041323776 | 2.869441408 | 11.40064312 | 12.46306463 | 12.418584 | 11.01049415 | 9.798659504 | 10.86440293 | SNAR-C1 |
| ENST00000423530 | 0.005526402 | 0.349265454 | 3.557746335 | 3.447178613 | 3.2766724 | 4.972847789 | 5.208318563 | 4.60389882 | LINC00607 |
| ENST00000614150 | 0.01890866 | 0.349911748 | 2.678957788 | 3.745389854 | 3.2408538 | 4.887230311 | 5.029810644 | 4.418488323 | RP11-234G16.5 |
| lnc-RP3-377D14.1.1-4:1 | 0.038993653 | 0.352575 | 3.382241436 | 2.615320763 | 3.4183166 | 3.881613723 | 5.150395757 | 4.755965558 | --- |
| lnc-SGK2-1:1 | 0.035503 | 2.819585957 | 6.268255114 | 7.122648793 | 5.9264196 | 4.427748216 | 5.086381452 | 5.420709801 | --- |
| NR_004383 | 0.027455141 | 2.816618608 | 7.575942145 | 8.068710097 | 6.9246451 | 6.570726446 | 5.764776042 | 5.826995703 | SNAR-G1 |
| lnc-PARVG-3:1 | 0.029198877 | 2.81473417 | 3.2553579 | 3.897867071 | 3.4266585 | 1.707684885 | 1.526656234 | 2.669274578 | --- |
| lnc-RABL2A-5:3 | 0.003353764 | 2.81275214 | 3.499415774 | 3.209346039 | 3.8218366 | 2.298713507 | 1.774043008 | 1.998649968 | --- |
| lnc-ZNF565-1:1 | 0.033904213 | 0.357185776 | 1.059957313 | 1.675796213 | 2.3567119 | 3.216850343 | 2.909930899 | 3.621558397 | --- |
| lnc-ZFHX4-5:1 | 0.030800205 | 2.784846809 | 5.26891695 | 5.617738253 | 5.1895599 | 4.467239622 | 3.361026831 | 3.607339665 | --- |
| ENST00000609032 | 0.016887864 | 0.361089292 | 3.50786734 | 2.73687285 | 2.8064541 | 4.873197018 | 3.952746268 | 4.61745927 | GS1-304P7.3 |
| lnc-SHC3-4:1 | 0.036748173 | 0.362208181 | 2.918920509 | 1.707922393 | 1.846232 | 3.518948907 | 4.111329895 | 3.469772127 | --- |
| ENST00000585867 | 0.044997095 | 0.362312024 | 3.173562398 | 2.202971741 | 3.0676639 | 4.018893866 | 4.969843864 | 3.722782581 | RP11-420N3.2 |
| lnc-SBF2-1:2 | 0.016739875 | 2.739209788 | 3.875516331 | 4.17606643 | 4.8067361 | 2.93231987 | 3.192860587 | 2.432278508 | --- |
| lnc-NCKAP1-5:1 | 0.031534989 | 2.726880515 | 3.52626216 | 3.391621937 | 4.4187146 | 2.574997399 | 2.71633431 | 1.759785197 | --- |
| ENST00000552524 | 0.000581845 | 0.366942953 | 2.023483618 | 2.057282858 | 2.2207497 | 3.455433885 | 3.735495701 | 3.437770823 | RP11-850F7.7 |
| ENST00000517495 | 0.032288516 | 0.367930148 | 4.365308533 | 3.139167219 | 3.7428068 | 5.221197323 | 5.591862029 | 4.945603126 | CTD-3080F16.3 |
| ENST00000607970 | 0.038854261 | 0.368445484 | 3.763404256 | 4.343993152 | 3.2039276 | 4.903723345 | 5.876669871 | 4.822482314 | RP11-395N3.2 |
| lnc-FSHR-4:1 | 0.018195084 | 2.698575806 | 5.273430143 | 6.056833156 | 5.7748929 | 4.735020191 | 3.744585528 | 4.26731539 | --- |
| lnc-ZNF582-5:3 | 0.045518997 | 0.370653417 | 5.967079282 | 5.596742306 | 4.6046784 | 7.35368684 | 6.622399131 | 6.684985089 | --- |
| lnc-ARSA-1:1 | 0.006066999 | 0.371691238 | 1.904426207 | 2.28449936 | 1.6379075 | 3.569699553 | 3.373285516 | 3.219730737 | --- |
| ENST00000529325 | 0.005561384 | 0.372060676 | 2.81976713 | 2.873295161 | 2.2816037 | 4.059083631 | 4.412403681 | 3.784051747 | RP11-513D5.5 |
| lnc-MYOZ2-1:1 | 0.036610171 | 2.687666149 | 6.633640143 | 7.276437731 | 7.0542302 | 5.912522317 | 4.772086174 | 5.820653881 | --- |
| ENST00000608405 | 0.03069189 | 0.374033792 | 3.257536291 | 2.762504358 | 2.0639909 | 4.209518177 | 4.523310264 | 3.743402225 | AP001412.1 |
| lnc-AC026150.9.1-2:2 | 0.041063043 | 0.3755999 | 6.215233326 | 6.797318956 | 7.5493652 | 8.599343791 | 8.576717964 | 7.800876394 | --- |
| NONHSAT093786 | 0.008915866 | 2.651627525 | 5.130634761 | 5.737581193 | 5.5191937 | 3.868080797 | 3.764391689 | 4.487880255 | --- |
| lnc-CEP112-2:1 | 0.013152247 | 0.377770623 | 2.065378453 | 2.677435927 | 2.0176144 | 3.184610167 | 4.016336646 | 3.750344833 | --- |
| lnc-PLEKHD1-3:1 | 0.043737724 | 2.624625767 | 6.849771354 | 7.763552626 | 7.322354 | 5.490488695 | 5.435150061 | 6.63655478 | --- |
| NONHSAT081070 | 0.017229275 | 0.381544385 | 1.873357815 | 2.477078138 | 2.7192357 | 3.961924244 | 3.800210117 | 3.57779258 | --- |
| ENST00000413564 | 0.005335302 | 2.619700057 | 4.765826497 | 4.515096566 | 4.4086125 | 3.485463753 | 2.862080886 | 3.129209497 | RP11-328K22.1 |
| lnc-POTED-11:1 | 0.008553111 | 0.381751582 | 3.957298645 | 3.832962456 | 3.4756634 | 5.032715553 | 5.53357155 | 4.812801336 | --- |
| lnc-FAM176A-3:1 | 0.018184714 | 0.382116926 | 5.828752298 | 5.924043389 | 6.4204965 | 7.497659642 | 7.845713129 | 6.919366967 | --- |
| lnc-OTX1-7:1 | 0.038422875 | 0.382155818 | 6.018778612 | 5.116728721 | 5.0564495 | 7.238158855 | 6.979811069 | 6.136242869 | --- |
| lnc-APBB1IP-1:4 | 0.029382502 | 0.382830765 | 2.807786567 | 1.949246303 | 2.8073455 | 3.306304372 | 4.275644047 | 4.121139213 | --- |
| lnc-LMO7-2:3 | 0.038376483 | 2.576686808 | 6.200290467 | 5.039950486 | 5.7267548 | 3.925231148 | 4.553744133 | 4.534763295 | --- |
| lnc-HOXC4-4:4 | 0.048032121 | 0.388480892 | 3.805838379 | 4.044440945 | 2.8877379 | 4.525991095 | 5.596244795 | 4.706542463 | --- |
| lnc-CR381653.1-2:1 | 0.025727531 | 0.388785641 | 2.417785739 | 2.255864995 | 3.2054022 | 3.532635666 | 4.451816642 | 4.026587032 | --- |
| NR_104199 | 0.020275702 | 2.5711185 | 3.243089233 | 4.114868845 | 3.9335934 | 2.720934765 | 2.246636542 | 2.32926864 | TSPAN16 |
| lnc-WRNIP1-5:1 | 0.007548658 | 0.390630171 | 2.625040336 | 3.040974915 | 2.4882524 | 4.190480891 | 3.669299576 | 4.339522401 | --- |
| ENST00000620994 | 0.044999929 | 0.391079098 | 2.349855687 | 2.065965846 | 1.7576244 | 2.667531905 | 3.732901213 | 3.671157225 | DLEU2_6 |
| ENST00000384320 | 0.005574256 | 2.542132133 | 3.58657512 | 4.052007939 | 3.4432486 | 2.175390323 | 2.218442691 | 2.668688333 | SNORD51 |
| lnc-ROS1-3:1 | 0.035641012 | 0.394128226 | 6.586392698 | 6.835950412 | 5.8568155 | 8.043089822 | 8.107451213 | 7.141213416 | --- |
| lnc-IL12RB2-4:2 | 0.009935852 | 2.537244117 | 3.014336247 | 3.223776384 | 2.9584441 | 1.878302769 | 1.908883771 | 1.321046307 | --- |
| lnc-CGA-4:1 | 0.043356919 | 2.53681795 | 3.800144066 | 4.873040936 | 4.3654052 | 3.25540737 | 2.96738807 | 2.962981592 | --- |
| lnc-CRYBA4-7:2 | 0.047926714 | 0.397319719 | 3.415962902 | 4.210846951 | 3.7747347 | 4.517017721 | 5.738315115 | 4.983676124 | --- |
| lnc-DCAF4L2-3:1 | 0.015176807 | 0.400335509 | 6.214646656 | 6.200851165 | 6.5646462 | 7.592247138 | 8.031262665 | 7.239583043 | --- |
| NR_003262 | 0.018442407 | 0.401071698 | 3.694886134 | 2.827439517 | 3.4053225 | 4.75348414 | 4.961213467 | 4.192080688 | FDPSP2 |
| ENST00000623305 | 0.01332531 | 2.489163336 | 4.860802931 | 5.244543338 | 5.4869752 | 3.819227179 | 3.880908273 | 4.006576401 | CTD-2385L22.2 |
| lnc-ROPN1L-2:1 | 0.018340516 | 2.478588524 | 6.198849414 | 6.095486272 | 5.4151156 | 4.867237274 | 4.409272875 | 4.585271645 | --- |
| lnc-KCNG2-1:2 | 0.027540767 | 2.477845067 | 5.075150467 | 4.741152346 | 5.7176126 | 3.972613625 | 3.587495412 | 4.16312495 | --- |
| ENST00000623680 | 0.026921033 | 2.474592664 | 4.617598229 | 5.600852255 | 5.2551338 | 4.207637113 | 3.563764849 | 3.874174756 | C18orf15 |
| lnc-PLEKHA2-4:1 | 0.036339865 | 2.456842988 | 8.425198882 | 8.718254908 | 8.5633869 | 7.178295022 | 6.723530864 | 7.74686884 | --- |
| lnc-ERVW-1-2:1 | 0.027635911 | 0.407156901 | 2.04131795 | 2.024004129 | 1.1857874 | 2.853971281 | 3.248118831 | 3.162658293 | --- |
| lnc-PRELID2-1:5 | 0.001358581 | 0.407565221 | 4.435754073 | 4.428088555 | 4.6184281 | 5.575837747 | 5.848501845 | 5.927662173 | --- |
| ENST00000528204 | 0.042098456 | 0.408987566 | 5.253641279 | 5.506229198 | 5.0640364 | 6.413849154 | 7.084952916 | 6.026295175 | NAV2-IT1 |
| lnc-CARKD-2:1 | 0.039838251 | 2.441872536 | 10.7366697 | 11.14755079 | 11.779016 | 10.0876267 | 10.13930365 | 9.733081003 | --- |
| lnc-GFRA1-2:1 | 0.022748528 | 2.441250617 | 3.550334281 | 3.543330172 | 3.3499679 | 2.271441215 | 1.714787442 | 2.495885577 | --- |
| lnc-NOL11-4:3 | 0.003729494 | 2.440749881 | 5.598070172 | 5.116303109 | 5.5144034 | 4.13952343 | 3.850058287 | 4.3742849 | --- |
| lnc-VPREB1-3:1 | 0.032061021 | 0.410040512 | 2.27414707 | 2.358289381 | 1.7433013 | 3.80169695 | 2.79667105 | 3.532732625 | --- |
| lnc-SGSM3-5:1 | 0.018681743 | 2.43176285 | 9.450929782 | 9.319442716 | 10.102734 | 8.617247907 | 8.338921452 | 8.160994621 | --- |
| ENST00000623261 | 0.038512071 | 0.411827955 | 6.09904047 | 5.502628975 | 6.0023054 | 6.637079316 | 7.69986106 | 6.962795646 | RP11-1110F20.1 |
| lnc-SHOC2-3:2 | 0.014443157 | 0.411883029 | 3.046546937 | 3.109092725 | 2.4898483 | 4.275609038 | 4.474047393 | 3.706449265 | --- |
| lnc-GABPA-8:2 | 0.04184992 | 0.412241426 | 4.863056369 | 3.931883575 | 3.7220299 | 5.649548368 | 5.753865201 | 5.148458685 | --- |
| ENST00000436546 | 0.038854186 | 2.421914811 | 3.771485009 | 3.460432531 | 4.2070038 | 3.083365791 | 2.482481813 | 1.904874202 | LINC00671 |
| lnc-CHRDL2-6:1 | 0.010155526 | 0.413433686 | 4.23429199 | 3.771602738 | 3.7266127 | 5.347529915 | 5.418723156 | 4.759465922 | --- |
| lnc-PPIAL4C-4:3 | 0.03527982 | 0.414579266 | 3.894095777 | 2.987631668 | 3.3294919 | 4.723775938 | 4.650620589 | 4.791809036 | --- |
| lnc-TMEM144-2:1 | 0.037477641 | 2.410637461 | 5.354758841 | 6.361105608 | 5.5238948 | 4.602911785 | 4.81723826 | 4.143099313 | --- |
| lnc-LARP4-3:1 | 0.006208297 | 0.415330881 | 3.656382211 | 3.529813396 | 3.0663366 | 4.915537101 | 4.506677326 | 4.668080411 | --- |
| lnc-ABCA5-5:1 | 0.041411602 | 0.416249544 | 5.515001149 | 5.071832684 | 5.4493057 | 6.405989411 | 7.144178058 | 6.11628534 | --- |
| lnc-C6orf138-2:1 | 0.012808546 | 0.417186832 | 2.257579938 | 2.369748899 | 2.9835282 | 3.825213727 | 4.117372746 | 3.495572858 | --- |
| lnc-CCDC152-7:2 | 0.007034591 | 2.396665222 | 6.303839835 | 6.463967876 | 5.9414627 | 5.077646398 | 4.85788332 | 5.029965743 | --- |
| lnc-ASB13-3:2 | 0.002727504 | 0.417763816 | 1.671704506 | 2.139283716 | 2.0441286 | 3.326099702 | 2.980661928 | 3.339295965 | --- |
| NR_036540 | 0.022997867 | 0.41792427 | 5.546547108 | 4.697326182 | 4.8149785 | 6.578845884 | 6.137651588 | 6.233911701 | LINC00622 |
| lnc-CCDC17-5:1 | 0.038214178 | 0.418199456 | 3.155152969 | 2.352905291 | 3.3584668 | 4.131925353 | 4.487590677 | 4.17606643 | --- |
| lnc-SCAI-1:1 | 0.032954729 | 0.419730963 | 4.959905672 | 4.60033531 | 4.0415753 | 5.925525007 | 6.184613596 | 5.234925553 | --- |
| ENST00000559600 | 0.029702311 | 2.380346411 | 3.023330768 | 3.641965727 | 3.1393077 | 2.545231957 | 1.519886784 | 1.870648529 | RP11-718O11.1 |
| lnc-PBX1-3:1 | 0.028664536 | 0.420674661 | 6.553418976 | 6.318514671 | 5.7059357 | 7.339574845 | 7.939410743 | 7.018290203 | --- |
| lnc-AL590867.1-7:1 | 0.007736371 | 2.373672188 | 6.045665123 | 6.343452634 | 5.8877268 | 4.872771806 | 4.911999367 | 4.785428433 | --- |
| NONHSAT059328 | 0.013754747 | 2.370098885 | 3.17934262 | 3.454060461 | 3.2832518 | 2.279472088 | 1.645347717 | 2.192345337 | --- |
| lnc-MAMSTR-1:1 | 0.049386272 | 0.423602323 | 3.652743589 | 2.604077836 | 3.4911592 | 4.511020695 | 4.716018713 | 4.426683933 | --- |
| ENST00000613300 | 0.043907515 | 2.358227013 | 5.27605081 | 5.904345361 | 5.0896956 | 4.7514151 | 4.125521064 | 3.570392036 | CTD-3116E22.8 |
| ENST00000608397 | 0.040884023 | 0.424530663 | 2.736641047 | 1.647089024 | 2.0997645 | 3.484683061 | 3.651647467 | 3.234583273 | RP11-757A13.1 |
| lnc-RNMT-1:1 | 0.017562824 | 2.354026447 | 3.854555478 | 3.655907299 | 4.277081 | 2.207430581 | 2.791866593 | 3.034199728 | --- |
| lnc-ZNF846-1:1 | 0.019981134 | 0.425277961 | 4.004772927 | 3.620483085 | 3.1651013 | 4.977191324 | 4.979527047 | 4.627147914 | --- |
| lnc-ANKRD50-6:1 | 0.020661025 | 0.426579146 | 2.972067494 | 2.170856254 | 2.6847712 | 3.967140422 | 3.964151436 | 3.675361803 | --- |
| ENST00000624351 | 0.005754318 | 0.42700926 | 5.20712051 | 4.800466581 | 4.7674343 | 6.348555729 | 6.284963918 | 5.80955327 | RP11-182N22.9 |
| ENST00000602327 | 0.03950197 | 0.42770191 | 4.186271201 | 3.5164402 | 3.6260606 | 4.868641993 | 5.540427962 | 4.487491423 | RP11-571M6.18 |
| lnc-MCMBP-2:1 | 0.032400377 | 2.332744687 | 3.464863807 | 3.722885899 | 3.6169838 | 2.856822859 | 2.112329006 | 2.030343083 | --- |
| lnc-AGAP1-5:1 | 0.008129857 | 2.330335197 | 7.194338374 | 6.779374001 | 7.4070602 | 6.158245208 | 5.756482787 | 5.841489177 | --- |
| lnc-RGS9-12:1 | 0.013447729 | 2.32713412 | 3.469540249 | 3.578032811 | 3.8460571 | 2.75894042 | 2.027110201 | 2.385877444 | --- |
| lnc-AC006455.1-10:1 | 0.009486478 | 2.319100379 | 3.896668936 | 4.170809939 | 3.8217333 | 2.803098171 | 3.019982816 | 2.377147971 | --- |
| ENST00000453892 | 0.046855935 | 0.431547242 | 2.870746428 | 3.245353475 | 2.2611235 | 4.018834139 | 4.064898655 | 4.094701595 | MMP24-AS1 |
| lnc-EPHA1-2:1 | 0.010328226 | 2.315966228 | 3.368025209 | 3.189968602 | 3.7806224 | 1.918081082 | 2.171347759 | 2.596661071 | --- |
| ENST00000575612 | 0.035736922 | 0.432038167 | 4.289247975 | 4.519000855 | 3.5376698 | 5.286028361 | 5.623929924 | 5.204448899 | CTC-508F8.1 |
| lnc-PXDNL-4:1 | 0.037563363 | 2.313677736 | 7.669852443 | 7.817473537 | 8.6231946 | 6.521320798 | 6.952015884 | 7.132040622 | --- |
| lnc-C1orf220-4:1 | 0.044135964 | 0.43241499 | 4.564518271 | 4.035288904 | 3.8246718 | 5.341570316 | 5.853090935 | 4.757780557 | --- |
| ENST00000591577 | 0.019985227 | 2.308793775 | 3.986546046 | 4.224628839 | 4.0166308 | 3.10607367 | 2.415153 | 3.005314785 | RP11-165F24.3 |
| lnc-TBC1D19-3:1 | 0.023664107 | 0.435482766 | 3.373420016 | 2.98352823 | 2.6560358 | 4.1196713 | 4.656072551 | 3.790401935 | --- |
| lnc-RGMA-2:4 | 0.032500938 | 0.436097457 | 2.397878821 | 1.804370038 | 1.395397 | 3.114390625 | 2.847084447 | 3.36047681 | --- |
| ENST00000625115 | 0.029137749 | 2.276555795 | 7.23566048 | 6.577172645 | 6.6416977 | 6.127742862 | 5.527691626 | 5.167810037 | CH507-254M2.2 |
| lnc-LRRC52-8:1 | 0.019480624 | 0.440331205 | 6.556311809 | 6.576557711 | 5.8368277 | 7.410580716 | 7.871701847 | 7.284723561 | --- |
| lnc-RP11-24B21.1.1-2:7 | 0.046463912 | 0.442510234 | 2.303580746 | 3.244434717 | 3.1654202 | 3.65979228 | 4.617176346 | 3.969679658 | --- |
| ENST00000605044 | 0.029509531 | 0.442597562 | 4.192633869 | 4.336519612 | 3.8554559 | 5.251526384 | 5.732415862 | 4.828066847 | RP1-309F20.4 |
| ENST00000586625 | 0.031478619 | 0.442622473 | 3.064861875 | 3.300052817 | 3.080573 | 4.74303563 | 4.182859561 | 3.938181013 | RP11-262H14.3 |
| lnc-CNPPD1-1:1 | 0.028217205 | 0.442800751 | 3.401092344 | 3.172475923 | 3.5539283 | 4.894273936 | 4.608459038 | 4.053904408 | --- |
| ENST00000623843 | 0.040569054 | 0.443385173 | 3.472762146 | 2.549235051 | 2.5145199 | 4.023558765 | 4.468816362 | 3.669147489 | RP11-298I3.3 |
| lnc-EIF2C2-2:2 | 0.048550358 | 0.443392477 | 3.081844717 | 2.23684838 | 1.9504227 | 3.988738304 | 3.573271939 | 3.414762324 | --- |
| ENST00000617667 | 0.03376529 | 2.253240942 | 5.339937828 | 5.247831626 | 4.500033 | 4.085997149 | 3.661343094 | 3.930349624 | RP11-8P13.5 |
| lnc-RBM6-3:1 | 0.002074768 | 0.444268626 | 3.793189213 | 3.377849234 | 3.599868 | 4.726423207 | 4.645251018 | 4.925884568 | --- |
| NONHSAT020831 | 0.013626071 | 2.249492029 | 4.09733062 | 3.769581107 | 4.4770927 | 2.676583709 | 2.874282255 | 3.299622673 | --- |
| lnc-ABCA8-3:1 | 0.036993455 | 0.446631689 | 3.396155683 | 2.453379237 | 2.7141065 | 3.875181546 | 4.234614319 | 4.089776934 | --- |
| lnc-C8orf83-7:6 | 0.032837635 | 0.44691641 | 5.709137988 | 5.002474503 | 5.0208591 | 6.433954257 | 6.842907952 | 5.911013771 | --- |
| lnc-RHBDD2-2:1 | 0.048669769 | 2.235434714 | 4.020360652 | 4.021887624 | 4.7019313 | 2.951059887 | 2.515790514 | 3.66864487 | --- |
| NR_120564 | 0.011315061 | 0.447483295 | 3.473440949 | 3.415441623 | 3.1250549 | 4.342563911 | 4.851897536 | 4.247620887 | LOC101928865 |
| lnc-DMTF1-3:1 | 0.0235795 | 0.448327201 | 4.487697499 | 4.312535053 | 3.6676043 | 5.309198586 | 5.591393254 | 5.12284267 | --- |
| lnc-FAM158A-1:2 | 0.008795986 | 2.230381326 | 4.329152227 | 4.314311353 | 4.8511954 | 3.490858241 | 3.020618809 | 3.52554258 | --- |
| ENST00000608775 | 0.031681226 | 0.448537723 | 2.955035146 | 2.682283406 | 3.5686209 | 4.221827513 | 4.118533047 | 4.460843748 | RP11-324L17.1 |
| lnc-IMP4-7:1 | 0.045823985 | 2.228307866 | 10.01525402 | 10.34679552 | 9.3063545 | 8.729428069 | 8.440693819 | 9.132033908 | --- |
| lnc-NOC2L-2:4 | 0.031952921 | 2.224891642 | 4.625691376 | 4.557193582 | 4.5192602 | 3.300594895 | 3.03370007 | 3.801347461 | --- |
| ENST00000577544 | 0.029596361 | 2.220973626 | 4.426039945 | 3.737984138 | 4.2080458 | 2.975721969 | 3.049087254 | 2.975395261 | CTD-2653B5.1 |
| lnc-TGDS-1:2 | 0.02642244 | 0.451150155 | 2.977003877 | 3.451008171 | 2.7645808 | 3.812210342 | 4.668357562 | 4.110985578 | --- |
| lnc-FAM3B-3:2 | 0.040144503 | 2.21520106 | 6.065702913 | 5.548481391 | 5.9425074 | 4.740962749 | 4.096413391 | 5.140819593 | --- |
| lnc-C17orf108-2:1 | 0.016024484 | 0.451620636 | 4.540367342 | 3.866832496 | 4.2653725 | 5.456236521 | 5.674951962 | 4.973567501 | --- |
| lnc-CABLES2-3:1 | 0.00797427 | 2.212031294 | 4.150965693 | 3.803093371 | 4.3955172 | 3.277858023 | 2.861141951 | 2.784713706 | --- |
| lnc-AC005838.1-4:1 | 0.023446 | 0.452450409 | 3.502909626 | 3.384159295 | 3.5310359 | 4.800687398 | 4.771882527 | 4.208698721 | --- |
| lnc-CRTAM-3:1 | 0.040210488 | 2.206886401 | 3.34360238 | 4.255687625 | 3.6094311 | 2.851833044 | 2.59745622 | 2.463331238 | --- |
| lnc-VWF-3:3 | 0.043372129 | 0.454156907 | 4.269366012 | 3.471130176 | 3.543585 | 5.40926205 | 4.843931098 | 4.415513135 | --- |
| lnc-WFIKKN1-1:2 | 0.042730258 | 2.20121676 | 7.983436199 | 8.310917243 | 8.0170379 | 7.072118309 | 7.326758039 | 6.354447792 | --- |
| ENST00000447019 | 0.020435949 | 0.454797736 | 5.222879592 | 4.452430338 | 4.5903623 | 6.181202381 | 5.89400332 | 5.677835757 | AC019186.1 |
| lnc-SLFN13-1:1 | 0.007098759 | 0.455108691 | 5.637226222 | 5.451029147 | 5.4829883 | 6.461765184 | 6.91695481 | 6.566330759 | --- |
| lnc-USP9Y-7:1 | 0.013029412 | 0.455548968 | 2.973978071 | 2.347742245 | 2.5895085 | 3.556456597 | 4.151769087 | 3.596173731 | --- |
| lnc-SLC45A2-1:1 | 0.044644633 | 0.455816214 | 3.105683478 | 3.036086045 | 3.0869166 | 4.024568205 | 4.639173947 | 3.840700446 | --- |
| NR_121668 | 0.009384492 | 0.455953205 | 3.536566914 | 4.132887355 | 3.6382613 | 4.620054718 | 5.057268299 | 5.059600148 | LOC101929122 |
| lnc-MRPL41-1:1 | 0.008981166 | 2.192041168 | 3.324011188 | 3.011970396 | 3.0623258 | 2.195601255 | 2.121334306 | 1.64498359 | --- |
| ENST00000611960 | 0.023439882 | 0.456894838 | 5.521151898 | 5.445191206 | 5.0093963 | 6.519770199 | 6.79848865 | 5.983934333 | AL391001.1 |
| NR_028351 | 0.012939379 | 0.457528258 | 3.810599624 | 4.468485353 | 3.9989064 | 5.56218709 | 5.151462793 | 4.963883651 | FLJ33360 |
| lnc-NEK10-2:1 | 0.035490652 | 2.180530865 | 3.981260134 | 4.712914999 | 4.8064913 | 3.228179642 | 3.64290498 | 3.35626187 | --- |
| ENST00000623446 | 0.038692156 | 2.173001256 | 3.451008171 | 3.971223584 | 3.3024305 | 2.873155165 | 1.870648529 | 2.53775781 | RP1-34P24.3 |
| lnc-TMEM207-2:1 | 0.003854302 | 0.460438691 | 4.04930597 | 3.867151977 | 3.6793504 | 4.750394325 | 5.228788255 | 4.95694443 | --- |
| lnc-CCDC8-2:1 | 0.003653748 | 0.460863898 | 2.631832523 | 2.169303404 | 2.4927811 | 3.433600863 | 3.782426156 | 3.440582798 | --- |
| lnc-USP33-2:2 | 0.042509142 | 0.461203872 | 3.087589303 | 2.026006723 | 2.5090987 | 3.810470182 | 3.360662696 | 3.935740506 | --- |
| lnc-ROBO1-3:1 | 0.025743609 | 2.166546637 | 3.439225983 | 3.99436621 | 3.362367 | 2.423065749 | 2.601179689 | 2.505575941 | --- |
| lnc-SBDS-23:1 | 0.02751592 | 2.166503645 | 4.066481895 | 4.354143534 | 4.041198 | 2.900694688 | 3.463996232 | 2.648411088 | --- |
| lnc-ADC-6:1 | 0.039084656 | 0.463919073 | 3.128785398 | 3.506054141 | 2.5071136 | 4.481096615 | 4.292867212 | 3.772330147 | --- |
| ENST00000526623 | 0.044462203 | 0.464272626 | 1.966014769 | 2.853312805 | 2.9042191 | 3.685894935 | 4.006036753 | 3.481318304 | AP003068.23 |
| lnc-IKZF1-9:1 | 0.012980158 | 2.151392983 | 3.777754713 | 3.822140035 | 3.218496 | 2.290201599 | 2.818151444 | 2.414798383 | --- |
| lnc-SUMF2-15:1 | 0.012944049 | 0.465423089 | 2.096952857 | 1.905582558 | 2.5299709 | 3.337308004 | 3.124552702 | 3.435954741 | --- |
| lnc-ACYP2-1:1 | 0.024696081 | 0.465542777 | 7.87089183 | 7.629856862 | 7.8602341 | 8.678849398 | 9.28106415 | 8.627369401 | --- |
| ENST00000624162 | 0.046547179 | 0.46763683 | 3.901844531 | 3.581850787 | 3.7913661 | 4.547712698 | 5.34327934 | 4.535616778 | bP-21264C1.2 |
| lnc-INSRR-2:1 | 0.040201441 | 0.468085243 | 4.801843848 | 5.16468935 | 5.3055676 | 5.992041387 | 6.677587591 | 5.773414536 | --- |
| NR_039981 | 0.03796098 | 0.468106982 | 5.655333922 | 4.754309655 | 4.8400711 | 6.438315865 | 6.315214687 | 5.91072624 | LINC01004 |
| lnc-CCP110-2:1 | 0.015899916 | 0.468511051 | 3.496189889 | 3.585696459 | 2.9173979 | 4.296423634 | 4.758346237 | 4.257119856 | --- |
| ENST00000624121 | 0.03404399 | 0.468893829 | 4.115947774 | 4.430412481 | 3.7016846 | 5.465993541 | 5.374511815 | 4.645382139 | RP11-203H19.2 |
| ENST00000553396 | 0.013635705 | 2.131490816 | 3.049422402 | 3.24389638 | 3.4580128 | 2.477554493 | 2.149646656 | 1.798050515 | LINC00517 |
| lnc-WDR73-8:1 | 0.019120537 | 0.47045176 | 3.551439687 | 2.942599349 | 2.8838794 | 4.397631854 | 4.430056553 | 3.839853911 | --- |
| NR_106961 | 0.020333521 | 2.125534171 | 6.270779648 | 7.023443391 | 6.7115721 | 5.391989726 | 5.548594244 | 5.859115331 | MIR7111 |
| lnc-PCBP3-2:1 | 0.020728187 | 0.471362576 | 3.465815636 | 3.404198144 | 2.8313225 | 4.37662888 | 4.193571574 | 4.454777695 | --- |
| ENST00000413311 | 0.035087519 | 0.47136409 | 6.152817603 | 5.286120182 | 5.4793314 | 6.643371635 | 6.663552965 | 6.989012822 | AC093382.1 |
| lnc-ATXN7-15:1 | 0.014873209 | 0.471863378 | 3.244434717 | 3.268603616 | 3.043241 | 4.505550253 | 4.331117109 | 3.919845366 | --- |
| ENST00000438380 | 0.03850499 | 2.11885386 | 3.683878822 | 3.86036991 | 4.1097162 | 3.305288933 | 2.53775781 | 2.426469923 | RP11-175D17.3 |
| lnc-RNF149-3:1 | 0.028367131 | 2.109675529 | 5.486243372 | 5.30737582 | 5.9804902 | 4.640877896 | 4.520287297 | 4.462715111 | --- |
| lnc-CHODL-10:1 | 0.006432217 | 2.109591416 | 3.377377784 | 3.067804552 | 3.1502231 | 1.831650913 | 2.155871179 | 2.348579042 | --- |
| ENST00000606235 | 0.03306269 | 0.474503372 | 2.938812415 | 2.732174392 | 3.5911974 | 4.31801646 | 4.46544908 | 3.757466373 | RP11-363E6.4 |
| lnc-IKBIP-5:1 | 0.022498775 | 0.474927426 | 1.893297313 | 2.287017232 | 1.9837153 | 2.756616773 | 3.055762724 | 3.504945926 | --- |
| lnc-YSK4-1:6 | 0.047474531 | 0.476876612 | 3.060635547 | 3.211375487 | 2.9923597 | 3.906591232 | 4.613387193 | 3.820855636 | --- |
| lnc-HSD11B1L-1:3 | 0.041449519 | 2.096520682 | 3.794756296 | 4.717505772 | 4.1659669 | 2.99626916 | 3.471602676 | 3.114090658 | --- |
| NR_104626 | 0.032839897 | 2.095174296 | 5.315110627 | 4.79998307 | 5.6424121 | 4.454163827 | 4.019380094 | 4.170061695 | LOC101928370 |
| lnc-GNAQ-9:1 | 0.047446695 | 2.094552663 | 3.913237191 | 4.769364528 | 4.2910076 | 3.307870058 | 3.195448464 | 3.391771553 | --- |
| lnc-BICD1-1:1 | 0.039905816 | 0.47808632 | 5.109684883 | 4.329875532 | 4.9395627 | 5.837008074 | 5.87114277 | 5.972193444 | --- |
| lnc-DNASE1-3:1 | 0.021410717 | 2.089505643 | 4.273720187 | 4.125218974 | 4.5724196 | 3.038456219 | 2.987693287 | 3.683370579 | --- |
| ENST00000400023 | 0.036958734 | 0.478646455 | 7.010449034 | 7.328798176 | 6.7385558 | 8.259416718 | 8.388701673 | 7.544910527 | C22orf34 |
| lnc-MAP9-8:1 | 0.040363354 | 2.081802548 | 4.289287737 | 5.092392763 | 4.5233103 | 3.982464502 | 3.648269721 | 3.084274907 | --- |
| lnc-MTIF2-4:1 | 0.022432235 | 0.480546931 | 2.44622364 | 2.084496857 | 2.7077425 | 3.4196672 | 3.561874744 | 3.492028617 | --- |
| lnc-AC233263.1-5:1 | 0.021118669 | 0.480746731 | 2.886365775 | 2.272918497 | 2.1743856 | 3.463821393 | 3.833375106 | 3.253168128 | --- |
| NR_024443 | 0.012734218 | 2.079653487 | 5.808326868 | 5.666610138 | 6.0628025 | 5.065423962 | 4.427519838 | 4.833431612 | LOC100133920 |
| lnc-ANP32E-1:1 | 0.004459923 | 2.079330692 | 4.481881287 | 4.661667649 | 4.8009614 | 3.854892425 | 3.470662728 | 3.428957121 | --- |
| ENST00000451656 | 0.018497372 | 2.078938405 | 6.529236585 | 7.194832352 | 6.8496522 | 6.184995689 | 5.683094489 | 5.531131753 | RP11-549L6.3 |
| NR_126326 | 0.016250055 | 2.076962657 | 2.86635446 | 3.398918619 | 3.3495481 | 1.907627785 | 2.539087988 | 1.975737782 | IGF2BP2-AS1 |
| lnc-ASB17-2:1 | 0.009955624 | 2.075546779 | 3.789740147 | 3.232544126 | 3.687612 | 2.247913643 | 2.694892893 | 2.62640879 | --- |
| NR_107009 | 0.028729945 | 2.075416197 | 3.814554639 | 3.847391092 | 4.1782457 | 3.219165818 | 2.966220049 | 2.408880151 | MIR7855 |
| lnc-OR2AT4-4:1 | 0.010487408 | 0.482385383 | 5.515839739 | 5.747252607 | 5.1393848 | 6.456751876 | 6.7781567 | 6.351847099 | --- |
| lnc-ATP1A1OS-2:7 | 0.042774039 | 0.482671628 | 3.682586531 | 2.896044699 | 2.8764278 | 4.512874823 | 4.403505879 | 3.727313609 | --- |
| lnc-SLCO5A1-8:1 | 0.030681907 | 2.069916703 | 3.676971681 | 3.54387618 | 3.4296991 | 2.268314048 | 2.220919537 | 2.914563057 | --- |
| lnc-GNG5-6:1 | 0.020240989 | 0.483438845 | 5.531619758 | 4.850221905 | 5.4186853 | 6.53479381 | 6.151896961 | 6.322015155 | --- |
| lnc-PIP-2:1 | 0.047840718 | 2.066994944 | 3.917245664 | 4.896318624 | 4.4432926 | 3.168897276 | 3.231999165 | 3.79210883 | --- |
| lnc-ZRSR2-1:1 | 0.039151377 | 0.483984594 | 3.756957204 | 3.172101988 | 4.1253987 | 5.065492322 | 4.734944948 | 4.492024785 | --- |
| lnc-SPTLC1-2:1 | 0.004035511 | 2.064495444 | 5.263956334 | 5.638352532 | 5.5176296 | 4.645039548 | 4.188546868 | 4.4379291 | --- |
| lnc-BLMH-2:1 | 0.046807881 | 2.064054743 | 9.005079054 | 9.969871352 | 9.5216834 | 8.899852306 | 8.257115517 | 8.263492011 | --- |
| lnc-GAPVD1-6:1 | 0.012072314 | 0.484977111 | 3.518438047 | 4.102435766 | 3.9323416 | 5.093023366 | 4.818620123 | 4.816408393 | --- |
| lnc-C5orf58-2:1 | 0.007559265 | 2.059960414 | 3.938557533 | 4.149235335 | 4.0744924 | 3.272577858 | 2.839099916 | 2.89067652 | --- |
| lnc-RCAN1-2:1 | 0.022417851 | 2.059278157 | 5.296865359 | 5.585849752 | 5.9759459 | 4.760837051 | 4.491555265 | 4.546664296 | --- |
| lnc-LINC00273-23:1 | 0.025425278 | 2.056286184 | 5.524031418 | 5.866558386 | 5.3578639 | 4.8747956 | 4.077774985 | 4.612887168 | --- |
| lnc-APTX-3:1 | 0.02171724 | 2.055411981 | 5.983375303 | 5.942086586 | 6.4486159 | 5.16004584 | 5.075150467 | 5.075589819 | --- |
| lnc-ERP44-5:1 | 0.028402716 | 0.487357627 | 2.463910624 | 3.060871825 | 2.2564576 | 3.46780191 | 4.016928105 | 3.458683001 | --- |
| lnc-CRHR1-6:1 | 0.009031746 | 0.488024061 | 4.232136107 | 3.69621359 | 3.975074 | 4.714353907 | 5.054393886 | 5.240733676 | --- |
| ENST00000449215 | 0.020873818 | 2.048162422 | 3.007949011 | 3.657333219 | 3.4312005 | 2.212677896 | 2.72462653 | 2.038852596 | RP11-344F13.1 |
| ENST00000612883 | 0.008209631 | 0.488324955 | 3.495415464 | 3.071275645 | 3.3856541 | 4.402192042 | 4.588003913 | 4.045726224 | DLEU2_3 |
| NR_037884 | 0.04299395 | 0.488357533 | 3.520542018 | 3.095355929 | 3.1037149 | 4.220750422 | 4.705532741 | 3.793368081 | LOC100507053 |
| NR_030229 | 0.010462413 | 2.044440702 | 4.834748066 | 5.252300387 | 5.2890004 | 4.417934331 | 3.846905456 | 3.996393969 | MIR504 |
| NR_106873 | 0.020620527 | 2.043074571 | 4.41829671 | 4.718522556 | 5.1365573 | 3.739503586 | 3.517475739 | 3.978293169 | MIR6815 |
| lnc-OR10AB1P-1:1 | 0.02913605 | 2.041739836 | 3.580318158 | 4.265281856 | 4.074787 | 3.326962641 | 2.954793845 | 2.521574464 | --- |
| ENST00000578129 | 0.037243977 | 2.040992533 | 4.275979446 | 4.593085341 | 4.255745 | 2.853658447 | 3.340332989 | 3.73556887 | RP11-958F21.3 |
| lnc-IGF2-AS-4:1 | 0.009672281 | 2.04007488 | 4.306761914 | 4.60686292 | 4.5391353 | 3.721241035 | 3.163200358 | 3.445564394 | --- |
| lnc-WSB1-9:1 | 0.00792973 | 2.039160968 | 3.226059073 | 3.573674464 | 3.2143462 | 2.586954635 | 2.037582065 | 2.282538453 | --- |
| lnc-ANKRD34C-1:1 | 0.005472695 | 2.038393548 | 3.895238845 | 3.50336003 | 3.5007322 | 2.506737587 | 2.759039405 | 2.576069144 | --- |
| lnc-FBXO24-1:4 | 0.017335122 | 0.490650599 | 2.50509209 | 2.478035065 | 2.2131051 | 3.043340985 | 3.534443322 | 3.64895444 | --- |
| ENST00000615933 | 0.01922226 | 2.037859385 | 4.522611067 | 5.13110406 | 4.8294463 | 4.200559368 | 3.572268381 | 3.602437427 | NAMA_2 |
| lnc-MRPS6-4:2 | 0.02389967 | 0.49080662 | 2.265913581 | 2.20473935 | 2.1397264 | 2.882764198 | 3.285808615 | 3.464951899 | --- |
| lnc-IL17RE-2:5 | 0.038029501 | 2.03736895 | 5.377959718 | 6.256893512 | 5.7300449 | 5.086329598 | 4.448104298 | 4.817483984 | --- |
| lnc-PCDH18-3:2 | 0.037338215 | 2.036221948 | 4.155926123 | 4.533325786 | 4.3899765 | 3.766412237 | 2.888995312 | 3.233788449 | --- |
| lnc-AC083864.4.1-4:1 | 0.046998098 | 0.491421381 | 2.996612224 | 2.45423577 | 3.2900389 | 4.03287411 | 3.97683468 | 3.924411369 | --- |
| ENST00000417273 | 0.043334263 | 2.034115757 | 3.751772908 | 3.5659602 | 3.5569689 | 2.82564022 | 2.06678588 | 2.797396723 | RP3-323N1.2 |
| lnc-C21orf119-4:1 | 0.027435702 | 2.033254549 | 4.331272958 | 5.055583526 | 4.6098152 | 3.544883538 | 3.61167713 | 3.845009463 | --- |
| NONHSAT057082 | 0.043209005 | 2.030458918 | 3.489851756 | 3.652964701 | 3.702374 | 2.855617529 | 2.064150936 | 2.748299302 | --- |
| lnc-DOCK4-6:1 | 0.032303866 | 0.492915556 | 4.229655168 | 3.965921751 | 4.1863592 | 5.291105033 | 5.383599201 | 4.689161408 | --- |
| lnc-RBMX2-1:2 | 0.024317704 | 0.493134781 | 2.298080275 | 2.999557907 | 2.442109 | 3.838642561 | 3.765997301 | 3.217152081 | --- |
| ENST00000621563 | 0.039904582 | 2.027077181 | 3.377699432 | 3.91183824 | 4.2610534 | 3.157946783 | 2.769220387 | 2.648161532 | uc_338 |
| lnc-GRASP-1:1 | 0.025971865 | 2.025923947 | 5.477713229 | 5.587928529 | 5.9678475 | 5.099984168 | 4.398949393 | 4.408592613 | --- |
| NR_046730 | 0.013551019 | 0.495166252 | 2.894077275 | 2.343496798 | 2.6710085 | 3.397127615 | 3.986845347 | 3.552647732 | AGBL5-AS1 |
| lnc-AL137145.1-7:1 | 0.025997637 | 0.495346358 | 3.534850153 | 2.762285994 | 2.9905021 | 3.820599368 | 4.420799613 | 4.136835029 | --- |
| ENST00000624661 | 0.021794119 | 0.495697683 | 5.292089801 | 4.865733436 | 4.8513976 | 6.16127304 | 6.258111272 | 5.584671829 | RP13-638C3.5 |
| ENST00000610430 | 0.021422027 | 0.495853329 | 1.84208244 | 2.545695615 | 1.9753276 | 3.302172085 | 2.859157622 | 3.295159532 | RP1-101A2.1 |
| ENST00000569713 | 0.043800559 | 2.016044531 | 6.026103793 | 6.193712462 | 5.7542112 | 5.482110653 | 4.716098488 | 4.610220107 | CASC22 |
| NR_110840 | 0.019379103 | 2.0134455 | 3.508601901 | 3.949148191 | 3.2592158 | 2.596884323 | 2.306820814 | 2.824091541 | LOC101927136 |
| NR_104214 | 0.023537245 | 2.005194894 | 6.590938726 | 6.372448773 | 7.0733473 | 5.396109509 | 6.031151717 | 5.61613904 | EYA3 |
| lnc-SCAND1-7:1 | 0.014037417 | 2.004483344 | 3.885033659 | 3.951351346 | 4.1456507 | 3.064606573 | 3.213570163 | 2.649696335 | --- |
| lnc-RP3-377D14.1.1-7:16 | 0.021263942 | 0.498955783 | 4.201428855 | 3.640815505 | 4.181504 | 4.919455243 | 5.383848536 | 4.712639981 | --- |
| ENST00000569778 | 0.011247153 | 2.002372508 | 4.148661461 | 4.635814873 | 4.2951579 | 3.488525877 | 3.324708405 | 3.297889488 | RP11-249C24.10 |
|  |  |  |  |  |  |  |  |  |  |
